# Effect of a multicomponent intervention on adherence to the Mediterranean diet in older adults: a randomized controlled trial

**DOI:** 10.3389/fnut.2026.1918299

**Published:** 2026-08-20

**Authors:** Ana María Murillo-Zaldívar, Rosario Alonso-Domínguez, Jesús González-Sánchez, Teresa Vicente-García, José Ignacio Recio-Rodríguez, Fausto José Barbero-Iglesias, María Luz Sánchez-Tocino, Laura García-Barrueco, María García-Oterino, Susana González-Manzano

**Affiliations:** 1 Complejo Asistencial Universitario de Salamanca, Instituto de Investigación Biomédica de Salamanca (IBSAL), Salamanca, Spain; 2 Departamento de Enfermería y Fisioterapia, Facultad de Enfermería y Fisioterapia, Instituto de Investigación Biomédica de Salamanca, Atención de Enfermería y Fisioterapia en promoción de la salud, estilos de vida y discapacidad, Universidad de Salamanca, Salamanca, Spain; 3 Gerencia de Atención Primaria de Salamanca, Salamanca, Spain; 4 Grupo de Investigación en polifenoles, Departamento de Química Analítica, Nutrición y Bromatología, Facultad de Farmacia, Instituto de Investigación Biomédica de Salamanca, Universidad de Salamanca, Salamanca, Spain

**Keywords:** diet, elderly nutrition, food and nutrition education, food labeling, healthy, Mediterranean diet, nutrition programs, older adults

## Abstract

**Background:**

The Mediterranean diet (MD) is one of the dietary patterns with the strongest evidence for preventing chronic diseases and promoting healthy aging. However, adherence to it is declining among the older population in Spain, in parallel with the rise in Westernized eating patterns. In this context, community-based interventions and the development of culinary skills are emerging as promising strategies for improving dietary quality and promoting healthy habits.

**Objective:**

To evaluate the effect of a multi-component intervention compared with a traditional group educational talk, on adherence to the MD and clinical parameters in people aged 60 and over.

**Methods:**

An experimental, randomized, controlled study was conducted with two parallel groups, in adults aged 60 years and older in Salamanca. The main variable was adherence to the MD and clinical parameters and sociodemographic variables such as secondary variables.

**Results:**

The sample comprised 130 participants. Linear mixed-effects modeling demonstrated a significantly greater improvement in MEDAS score over time in the intervention group than in the control group (group × time interaction: *p* = 0.027). Descriptively, the intervention group showed particularly favorable improvements in their consumption of extra virgin olive oil, fruit, vegetables and pulses, alongside a reduction in ultra-processed foods and sugary drinks.

**Conclusion:**

The multi-component intervention was more effective than a traditional educational talk in improving adherence to the Mediterranean diet among community-dwelling older adults. Improvements were maintained over the three-month follow-up period.

## 1 Introduction

The Mediterranean diet (MD) is characterized by a high intake of plant-based foods, including fruit and vegetables (FV), whole grains, pulses and nuts; a moderate intake of fish, lean meats, eggs and dairy products; and a low intake of red meat and saturated fats. Extra virgin olive oil (EVOO), the main fat source in the Mediterranean diet, is the preferred form of olive oil consumption due to its higher polyphenol content and the health benefits associated with it (1–3). This dietary pattern has a distinctive nutritional profile, with a high density of micronutrients and bioactive compounds, including vitamins, monounsaturated fatty acids, omega-3 (n-3) fatty acids, dietary fiber and a wide variety of phenolic compounds (polyphenols) with potential antioxidant and anti-inflammatory activity (2, 3).

Beyond its nutritional composition, the MD is underpinned by the consumption of fresh, locally sourced foods, as well as traditional culinary practices, the regular use of EVOO in home cooking, simple cooking techniques and the use of herbs and spices. These elements reinforce not only its nutritional value, but also its cultural dimension and sustainability (1).

Furthermore, the MD is recognized as one of the healthiest dietary patterns, with robust scientific evidence supporting its benefits for health and quality of life, particularly among older adults. High adherence to this pattern is associated with lower all-cause mortality and a reduced risk of cardiovascular disease (CVD) and other chronic non-communicable diseases, such as cancer, diabetes mellitus, obesity and metabolic syndrome, contributing to an overall reduction in morbidity and mortality. Furthermore, the MD has been associated with beneficial effects on inflammation, oxidative stress, immune function, gut microbiota and cognitive decline (4–8). In this context, high adherence to the MD is inversely associated with the risk of frailty and pre-frailty and directly associated with a longer disability-free life expectancy, reinforcing its role as a key strategy for healthy aging (4–6).

At the same time, population aging is a growing phenomenon worldwide (3). In Spain, over 20% of the population is aged 65 or over, with the figure reaching nearly 30% in the municipality of Salamanca, making it one of the areas with the highest aging rates (3, 9). Added to this demographic picture is the high prevalence of chronic non-communicable diseases, which are closely linked to the progressive shift away from traditional healthy dietary habits toward westernized dietary patterns, characterized by high intakes of sodium, trans fats and calories, and lower intakes of foods typically associated with diabetes (10). Consequently, poor dietary quality in older adults is associated with lower long-term survival, higher all-cause mortality and a greater overall burden of disease (2, 3, 5, 10–12).

Although older people recognize the importance of the MD, adherence to this dietary pattern continues to decline. In Spain, around 90% of people aged 65 and over have a diet that could be improved (10, 13), and only a minority achieve adequate levels of adherence.

Similarly, Navarro-Martínez et al. (14) found that only 23.7% of their sample of adults aged over 60 in the Valencian Community adhered adequately to the MD. These findings are consistent with data from the European Health Survey in Spain (15) and with reports from the Spanish Society of Community Nutrition (16), which show a high prevalence of overweight, low intake of fruit and vegetables, and a progressive shift toward dietary patterns of lower nutritional quality in the older population.

MD is one of the modifiable lifestyle factors with the greatest potential for intervention in older people and has become a standard component of health promotion strategies, particularly in multi-component programs aimed at changing various health-related behaviors. In this context, landmark trials such as PREDIMED (17) and PREDIMED-Plus (18) have consolidated the evidence on the effectiveness of the MD in the prevention and management of cardiometabolic risk factors.

However, most interventions aimed at improving adherence to the MD in older people continue to rely primarily on the provision of nutritional information, while few incorporate practical components, such as interpreting nutritional labeling, food selection or the development of cooking skills. This limitation is particularly significant, as eating behavior depends not only on nutritional knowledge but also on practical skills such as meal planning, food shopping, reading labels and cooking techniques, all of which are fundamental to maintaining sustained dietary changes.

In this regard, various studies have shown that interventions combining nutritional education with practical activities focused on food selection, menu planning and the acquisition of cooking skills lead to greater adherence to the Mediterranean diet, as well as improving diet quality, health indicators and the sustainability of dietary patterns (19, 20). Furthermore, educational programs aimed at older adults have proven effective in increasing both knowledge of and practical application of this dietary pattern, particularly when integrated into multifactorial interventions that include other lifestyle changes, thereby enhancing their benefits for health and quality of life (21).

Consistently, recent studies indicate that greater nutritional literacy and the development of cooking skills through practical workshops encourage healthier food choices, increase confidence in cooking, promote the consumption of fresh foods and reduce the intake of ultra-processed products, resulting in greater adherence to the Mediterranean diet (22–25). In contrast, a lower level of cooking skills has been associated with poorer diet quality, higher consumption of ultra-processed foods and lower adherence to healthy dietary patterns, including the MD.

Similarly, Casucci et al. (26), observed that, in adults, both dietary and culinary skills and health literacy relating to diabetes were independently associated with greater adherence to this dietary pattern, supporting the incorporation of strategies that combine practical training and nutritional education.

As well as promoting adherence, culinary skills can influence the nutritional value of the foods that characterize the MD diet. In particular, cooking methods and processing conditions can alter the content and bioavailability of phenolic compounds and other bioactive components present in extra virgin olive oil and plant-based foods, potentially reducing some of the health benefits associated with this dietary pattern.

Despite this evidence, there are still few multidisciplinary community-based interventions that integrate nutrition education with practical tools applied to everyday situations relating to food shopping, selection, labeling and preparation (27). Consequently, the combination of dietary education, nutritional literacy and the development of cooking skills represents a promising strategy for improving dietary quality and promoting greater adherence to the Mediterranean diet among older people.

Furthermore, the available evidence shows that most interventions aimed at improving adherence to the MD have been developed for very specific populations, such as children, students or older adults with diagnosed conditions, with few targeting older people living in the community who do not meet specific clinical criteria (28).

The aim of this study was therefore to evaluate the effect of a multi-component intervention, delivered in real-life settings involving food shopping and preparation (labeling, selection of fresh produce, a cooking workshop and an educational tasting session), compared with a traditional group educational talk, on adherence to the MD and various clinical parameters in people aged 60 and over.

## 2 Materials and methods

### 2.1 Study design

An experimental, randomized, controlled study was conducted with two parallel groups, involving eight associations for older people located in different neighborhoods of Salamanca (Spain), and two municipal centers for older people run by Salamanca City Council.

### 2.2 Study population/participants

From the total of 151 people participating in the “Research into Active Aging with Preventive Physiotherapy (PReGe)” (29) program, a project arising from the collaboration agreement between Salamanca City Council and the University of Salamanca, 130 were ultimately included in the study.

The inclusion criteria were: being aged 60 or over, being retired or in early retirement, residing in the municipality of Salamanca, being enrolled in the PreGe program, participating in the study after receiving the relevant information, and signing the informed consent form.

The exclusion criteria were: having a history of or current musculoskeletal conditions that limited mobility; suffering from a clinically demonstrable neurological and/or neuropsychological disorder; or failing to meet the inclusion criteria.

### 2.3 Randomization and masking

**Figure 1:**
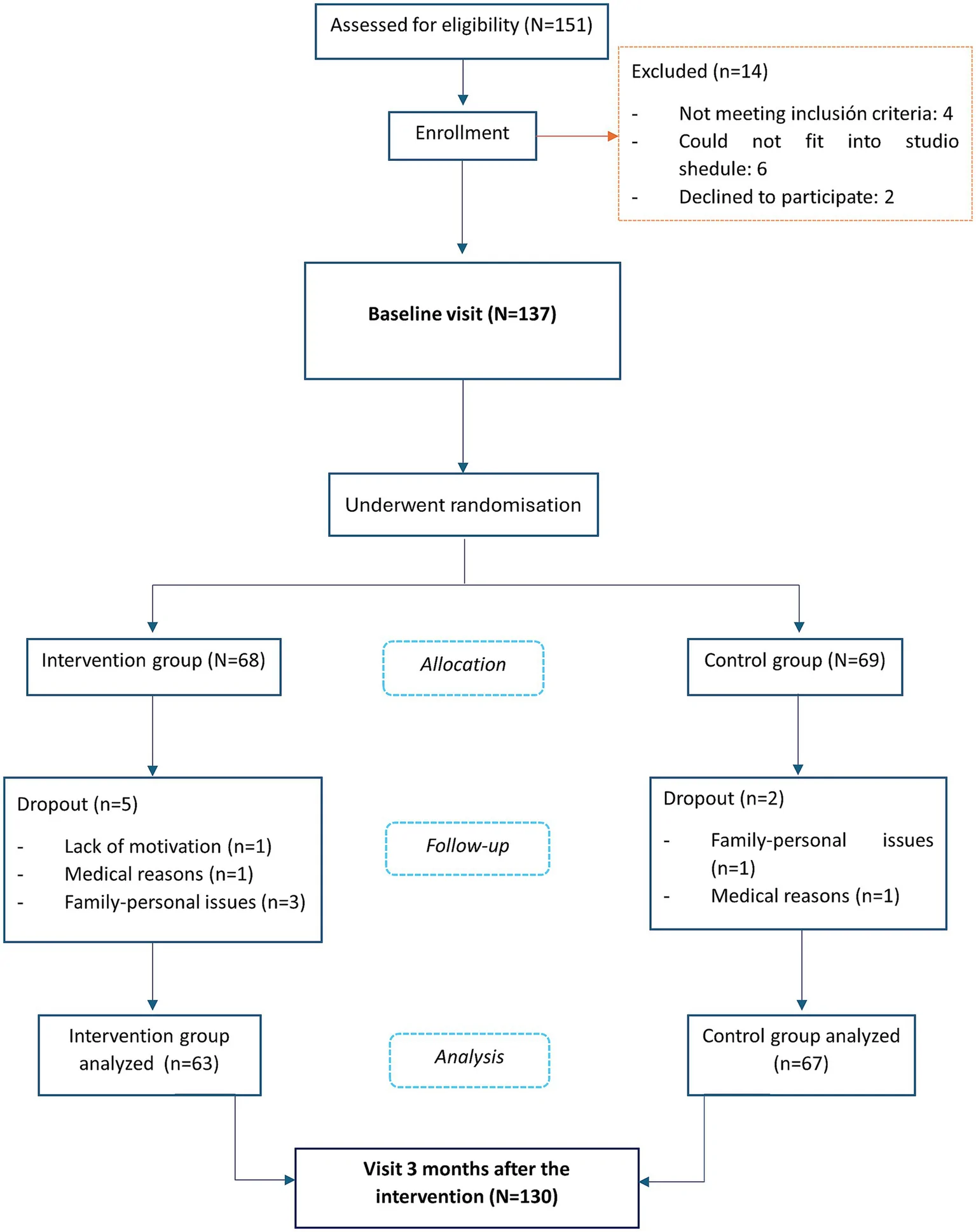
Study flowchart: participant enrolment and study completion.

Participants were allocated using a simple randomization procedure. After providing written informed consent and completing the baseline assessment, participants were randomly assigned in a 1:1 ratio to the intervention group (IG) (*n* = 63) or the control group (CG) (*n* = 67). The allocation sequence was generated by an independent researcher using the Epidat 4.0 programme (Consellería de Sanidade, Santiago de Compostela, Spain). The allocation sequence was concealed until group assignment and was not disclosed before allocation (Figure 1).

Due to the nature of the intervention, neither the participants nor the person responsible for delivering it could be blinded. However, the researchers responsible for outcome assessments at each study visit and those performing the statistical analyses remained blinded to group allocation.

As participants were recruited from several community centers, the potential for contamination between groups was considered during the study design. To minimize this risk, both groups received the same standardized educational session and general nutritional leaflet, whereas the intervention-specific practical workshops were delivered exclusively to participants allocated to the intervention group after randomization. Invitations to these workshops were made individually by telephone, and the workshop materials were distributed only to intervention participants. Furthermore, the workshops were conducted independently of the centers’ routine activities. Although informal interaction between participants attending the same community centers during the academic year cannot be completely excluded, no joint intervention sessions beyond the common educational component were conducted, thereby reducing the likelihood of contamination.

### 2.4 Calculation of sample size

The sample size was estimated using adherence to the MD as the primary variable, measured using the Mediterranean Diet Adherence Screener (MEDAS) questionnaire. The sample size calculation was based on a prespecified detectable between-group difference of 0.545 MEDAS and an assumed standard deviation of 0.88 points, which were defined *a priori* during the study design as the methodological assumptions for the sample size calculation. At the time the study was designed, no intervention studies directly comparable in terms of population, setting and primary outcome were available to support these assumptions with a specific published estimate. Assuming an expected dropout rate of 15%, a sample size of 126 subjects (63 in each group) was calculated. Finally, it was deemed sufficient to include 130 subjects to detect relevant differences in the study’s main variable.

### 2.5 Variables and measurement instruments

#### 2.5.1 Main measurement variable (nutritional assessment)

##### 2.5.1.1 Adherence to the Mediterranean diet

The main variable was adherence to the MD, assessed using the MEDAS questionnaire in its Spanish-translated and validated version (21, 30). This scale is a validated tool for the rapid assessment of adherence to the MD and comprises a total of 14 items. It includes 11 questions on the frequency of consumption of different food groups: portions of fruit and vegetables, consumption of butter, margarine and red meat, weekly consumption of wine, pulses, fish and seafood, nuts, pastries, and whether white meat is preferred over red meat. Two questions concern the consumption of popular foods used in Spanish cuisine: EVOO and wine. One question addresses the method of cooking or preparing dishes (sofrito). For each question regarding the quantity consumed, 1 or 0 points are awarded, resulting in a total score between 0 and 14 points. A total score of 9 or above was considered indicative of adequate adherence to the MD.

#### 2.5.2 Secondary variables

Clinical parameters and sociodemographic variables were recorded as secondary variables.

Among the non-anthropometric clinical parameters, systolic blood pressure (SBP) and diastolic blood pressure (DBP) were assessed. Three measurements were taken, and the average of the last two was used. An OMRON M10 blood pressure monitor, validated in accordance with the protocol of the European Society of Hypertension was used for this purpose (31, 32).

Anthropometric parameters recorded included weight, height, body mass index (BMI) and waist circumference. Weight was measured with participants barefoot and wearing light clothing. Height was determined using a portable system (Seca 222), recording the average of two measurements and rounding to the nearest centimeter. BMI was calculated as weight in kilograms divided by height in meters squared. Waist circumference was measured using a flexible tape measure, held parallel to the floor, in accordance with the recommendations of the Spanish Society for the Study of Obesity (32).

Sociodemographic variables were also collected, including age, sex, marital status and educational level.

### 2.6 Intervention

To ensure intervention, fidelity and reproducibility, all sessions were delivered by the same trained multidisciplinary team following a standardized teaching protocol, with identical educational materials and procedures across all participants.

#### 2.6.1 Common intervention in both groups

Before randomization, all participants received the same standardized nutritional education intervention. This consisted of a group educational session, delivered in groups of 10–12 participants by healthcare professionals with expertise in nutrition (nurse and/or pharmacist), using a predefined educational protocol and standardized teaching materials to ensure consistent delivery across all study groups. The educational content was based on current Mediterranean diet recommendations and covered healthy eating in older adults, the Mediterranean dietary pattern and the “5S” healthy eating model, the Mediterranean food pyramid, food groups and recommended consumption frequencies, the nutritional properties and health benefits of the main food groups, healthy cooking techniques, the Harvard Healthy Eating Plate method, and practical examples of balanced menus. A standardized PowerPoint presentation was used throughout the session, and all participants received a printed educational leaflet containing the key concepts and general nutritional recommendations to reinforce understanding and facilitate adherence to the Mediterranean diet (Supplementary Figure S1).

#### 2.6.2 Specific intervention by the intervention group

The intervention group also took part in a multi-component intervention comprising two activities, which are described below. For each activity, they were given a leaflet containing key information to take home, developed specifically for the project (Supplementary Figure S2). In addition to the standardized educational session, participants allocated to the intervention group received two practical workshops designed to reinforce the acquisition of knowledge through experiential learning.

##### 2.6.2.1 Shopping basket workshop

The first workshop focused on healthy food purchasing, was led by two specialists in nursing and nutrition at a municipal market and a supermarket in Salamanca, with groups of 8–10 participants per session. The content was based on current recommendations regarding MD, information on food nutrition labeling (list of ingredients, nutrition table and the Nutri-Score system), as well as guidance on selecting foods with the best nutritional composition to optimize the shopping basket. During the session, appropriate portion sizes for different food groups were discussed, with particular emphasis on adhering to recommendations for the consumption of fruit and vegetables, pulses, nuts, dairy products and EVOO as the main cooking fat, as well as prioritizing whole grains over refined grains. It is also distinguished between foods for regular consumption and those for occasional consumption, highlighting the need to limit ultra-processed products with high levels of added sugars, saturated fats or salt. The session lasted approximately 90 min.

##### 2.6.2.2 Preparing and tasting a healthy meal

The second practical workshop consisted of the supervised preparation of a healthy three-course Mediterranean menu by the participants. Lasting approximately 90 min, it was designed to reinforce the knowledge acquired during the educational session and the healthy food purchasing workshop through the practical application of Mediterranean diet recommendations. Participants prepared dishes using healthy ingredient selection and cooking techniques aimed at reducing the use of salt, added sugars, and saturated fats. Throughout the session, key concepts related to recommended food group proportions, portion sizes, and healthy meal planning were reinforced. Finally, the activity concluded with a guided tasting session in which participants evaluated the sensory characteristics and acceptability of the dishes, promoting confidence in the feasibility and enjoyment of healthier culinary practices. The main objective of this intervention was to reduce the salt and added sugars and saturated fats intake of the participants while maintaining palatability using herbs, spices, and other flavors enhancing alternatives.

### 2.7 Structure of the visits

The recruitment coordinator contacted those enrolled in the PReGe Programme by telephone. Each participant completed two assessment visits: firstly, a baseline visit at the start of the study (prior to randomization) and a follow-up visit 3 months after the first. Both visits followed a similar structure and lasted approximately 60 min.

- Baseline visit: The variables described above were recorded, and participants were informed of the agreed date for the standardized counseling session, which was the same for both groups. Randomization was then carried out, and participants in the IG were contacted to inform them of the dates of the specific activities.
- 3-month follow-up visit: This was similar to the baseline visit and allowed for the re-assessment of the study variables.

### 2.8 Statistical analysis

Results were expressed as the mean ± standard deviation for quantitative variables or as a frequency distribution for qualitative variables. The results were analyzed on an intention-to-treat basis. Normality was assessed using the Kolmogorov–Smirnov test. Baseline characteristics were compared between groups using Student’s *t*-test for continuous variables and the chi-square test for categorical variables. Changes in individual MEDAS items were analyzed exploratorily using McNemar’s test for within-group comparisons and the chi-square test for between-group comparisons. The primary outcome (MEDAS total score) was analyzed using linear mixed-effects models including group (intervention/control), time (baseline/3 months) and the group x time interaction as fixed effects, with participant included as a random intercept and an unstructured covariance matrix. Two models were fitted: an unadjusted model and a model adjusted for age and sex. The group x time interaction was considered the primary estimate of the intervention effect. The same modeling strategy was applied to secondary continuous outcomes (body mass index, systolic blood pressure and diastolic blood pressure). Estimated marginal means were obtained from the unadjusted model for graphical representation, and adjusted effect estimates with 95% intervals were reported. For all analyses, statistical significance was established at two-sided *p* < 0.05. The statistical software used was SPSS, v.28.0.

### 2.9 Ethical considerations

The study was approved by the Ethics Committee for Clinical Trials of the Salamanca Health District on 13 May 2024 (PI 2024 051674).

Participants will be asked to sign an informed consent form after receiving the study information in accordance with the Declaration of Helsinki (33). Confidentiality will be guaranteed in accordance with the provisions of Organic Law 3/2018 of 5 December on the Protection of Personal Data and the Guarantee of Digital Rights, and Law 14/2007 on biomedical research.

## 3 Results

### 3.1 Baseline characteristics of the study population

The sociodemographic and anthropometric characteristics of the study population are shown in Table 1. The sample comprised 130 people aged over 60 living in Salamanca, of whom 109 were women (83.8%). No statistically significant differences were observed between the CG and the IG in the baseline sociodemographic and anthropometric variables, suggesting adequate comparability between the two groups at the start of the study. Only diastolic blood pressure (DBP) was significantly higher in the IG than in the CG (*p* = 0.038), while systolic blood pressure (SBP) showed a trend toward significance (*p* = 0.053).

**Table 1:** Sociodemographic and anthropometric characteristics of the study population.

Variables	Control group (67)	Intervention group (63)	*P*-value
Age (years), mean (SD)	76.1 ± 5.6	75.9 ± 6.4	0.840
Gender, *n* (%)			
Female	58 (86.6)	51 (81.0)	0.385
Male	9 (13.4)	12 (19.0)	
Civil status, n (%)			
Married	33 (48.5)	36 (57.1)	0.946
Separated	7 (10.3)	3 (4.8)	
Widowed	23 (33.8)	12 (19.0)	
Single	4 (5.9)	10 (15.9)	
Other	0	1 (1.6)	
Educational level, *n* (%)			
Elementary school	46 (67.6)	32 (50.8)	0.309
Middle or high school	12 (17.6)	24 (38.1)	
University studies	8 (11.8)	3 (4.8)	
Doctorate	0	1 (1.6)	
Systolic blood pressure (mmHg), mean (SD)	129.4 ± 18.0	136.2 ± 19.8	0.053
Diastolic blood pressure (mmHg), mean (SD)	78.7 ± 9.1	82.4 ± 10.0	0.038
Waist circumference (cm), mean (SD)	92.1 ± 11.9	92.3 ± 9.7	0.940
BMI, mean (SD)	26.2 ± 6.5	27.0 ± 5.4	0.506

Values are expressed as mean ± standard deviation (SD) for quantitative variables, and as absolute and relative frequencies (%) for categorical variables. SD, Standard Deviation; BMI, body mass index.

### 3.2 Changes in Mediterranean diet adherence after the intervention

**Figure 2:**
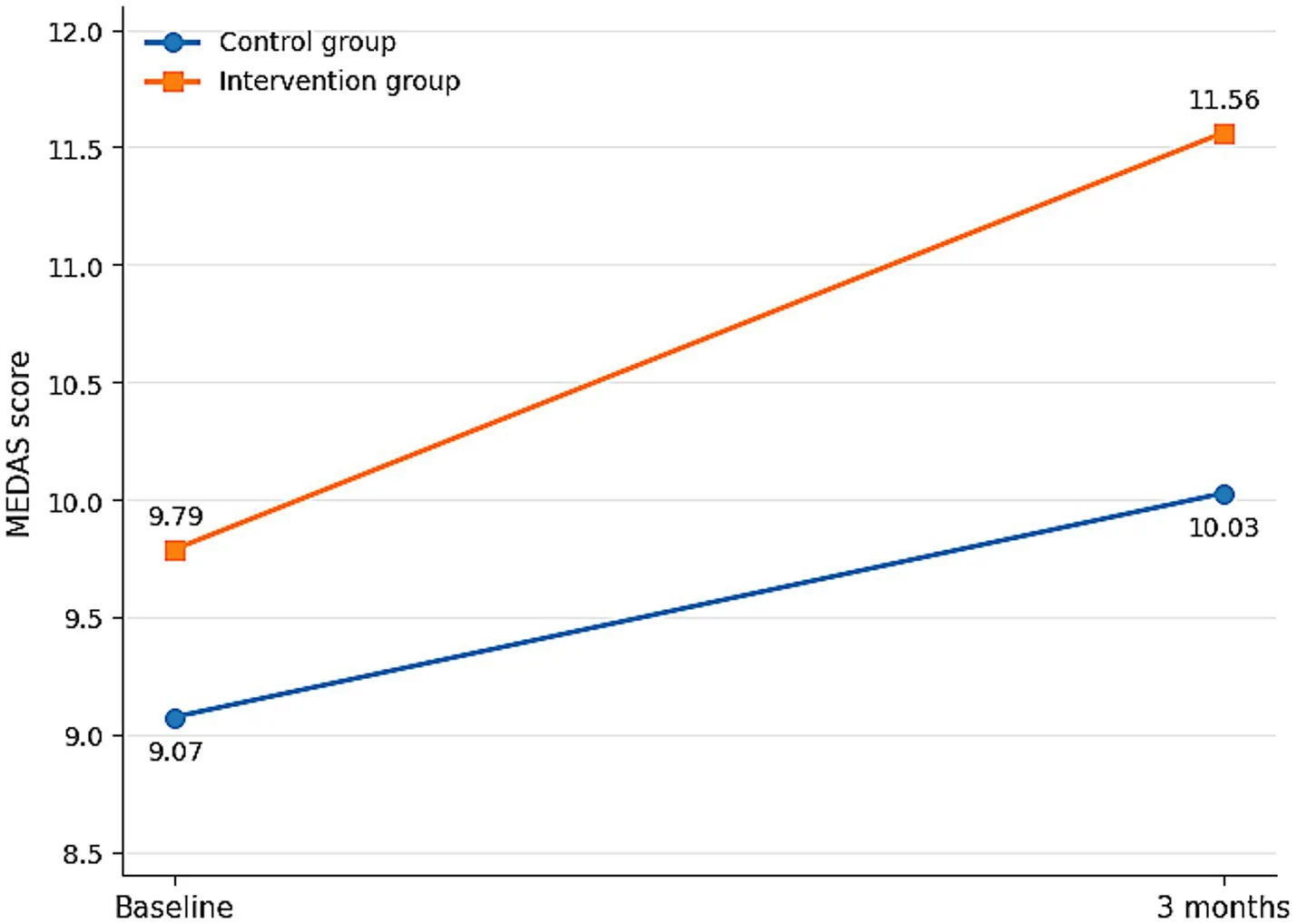
Estimated marginal means of MEDAS score according to study group obtained from the unadjusted linear mixed-effects models.

Changes in adherence to the Mediterranean diet were analyzed using linear mixed-effects models (Table 2). A significant group x time interaction was observed in the unadjusted model (*F* = 5.415, *p* = 0.022), indicating that changes in MEDAS score differed between the intervention group and control groups over time. This finding remained significant after adjustment for age and sex (*F* = 5.003, =0.027). The adjusted model estimated a between-group difference change of 0.804 MEDAS points in favor of the intervention group (95% CI 0.092–1.516; *p* = 0.027). Estimated marginal means from the unadjusted model are presented in Figure 2.

**Table 2:** Linear mixed-effects models evaluating changes in MEDAS score before and after the intervention.

Fixed effect	Unadjusted F	*p*-value	Adjusted model F (df)	*p*-value
Group	15.604	<0.001	18.545	<0.001
Time	59.714	<0.001	56.410	<0.001
Group × Time	5.415	0.022	5.003	0.027
Sex		–	2.821	0.096
Age		–	0.558	0.456

Linear mixed-effects models included group, time and the group × time interaction as fixed effects and participant as a random intercept with an unstructured covariance matrix. The adjusted model additionally included age and sex as covariates.

An exploratory analysis including a Group × Time × Sex interaction showed no evidence that the intervention effect differed according to sex (Group × Time × Sex interaction, *p* = 0.839).

### 3.3 Adherence to individual Mediterranean diet criteria

To identify which dietary behaviors contributed to the overall improvement in Mediterranean diet adherence, the individual MEDAS criteria were analyzed before and after the intervention. Baseline adherence to the individual MEDAS criteria is presented in Table 3.

**Table 3:** Baseline adherence to individual Mediterranean criteria.

Mediterranean diet criteria	Control group (67)	Intervention group (63)	*p*-value
Use olive oil as main culinary fat	63 (92.6)	60 (95.2)	0.536
Olive oil (4 tablespoons/day)	61 (89.7)	55 (87.3)	0.666
Vegetables (2 servings/day)	37 (54.4)	39 (61.9)	0.385
Fruits (3 servings/day)	55 (80.9)	51 (81.0)	0.992
Red or processed meats < 1 serving/day	42 (61.8)	51 (81.0)	0.016
Butter, cream or margarine < 1 serving/day	46 (67.6)	47 (74.6)	0.381
Sugar-sweetened beverage < 1 cup/day	47 (69.1)	44 (69.8)	0.928
Red wine ≥ 7 servings/week	18 (26.5)	12 (19.0)	0.312
Legumes ≥ 3 servings/week	31 (45.6)	34 (54.0)	0.338
Fish or seafood ≥ 3 servings/week	43 (63.2)	41 (65.1)	0.730
Commercial pastries (<2 servings/week)	22 (32.4)	29 (46.0)	0.109
Nuts ≥ 3 servings/week	36 (52.9)	49 (77.8)	0.003
White meats more than red meats	62 (91.2)	51 (81.0)	0.089
Sofrito ≥2 servings/week	54 (79.4)	55 (87.3)	0.227

Values are expressed as mean ± standard deviation (SD) for quantitative variables, and as absolute and relative frequencies (%) for categorical variables. Statistically significant differences between groups are indicated as follows: * (*p* < 0.05) difference between control group and intervention group.

Analysis of the individual items of the MEDAS questionnaire revealed some baseline differences between groups. Specifically, the IG had a higher proportion of participants with lower consumption of red or processed meat (<1 portion/day) compared with the CG (81.0% vs. 61.8%; *p* = 0.016) as well as a higher proportion of participants with adequate consumption of nuts (≥3 portions/week) (77.8% vs. 52.9%; *p* = 0.003) compared with the CG. Regarding the remaining items assessed, no statistically significant differences were observed. However, overall, it can be seen that although both groups showed broadly similar baseline adherence to the MD, the intervention group had more favorable scores on some specific items of the MEDAS questionnaire.

Changes in adherence to the individual MEDAS criteria after the intervention are shown in Table 4.

**Table 4:** Changes in adherence to individual Mediterranean diet criteria after the intervention.

Mediterranean diet criteria	Control group (67)	Intervention group (63)	*p*-value
Use olive oil as main culinary fat	2 (2.9)	3 (4.7)	0.623
Olive oil (4 tablespoons)	3 (4.4)	7 (11.1)	0.148
Vegetables (2 servings/day)	10 (14.9)	16 (25.3)	0.122
Fruits (3 servings/day)	5 (7.4)	8 (12.6)	0.333
Red or processed meats < 1 serving/day	15 (22.3)	8 (12.6)	0.115
Butter, cream or margarine < 1 serving/day	10 (14.9)	13 (20.6)	0.461
Sugar-sweetened beverage < 1 cup/day	10 (14.9)	13 (20.6)	0.460
Red wine (7 servings/week)	2 (2.9)	8 (12.6)	0.031
Legumes (3 servings/week)	9 (13.4)	13 (20.6)	0.286
Fish or seafood (3 servings/week)	8 (11.9)	7 (11.1)	0.889
Commercial bakery (2 servings/week)	12 (17.9)	18 (28.5)	0.125
Nuts (3 servings/week)	15 (22.3)	7 (11.1)	0.079
White meats more than red meats	5 (7.4)	9 (14.2)	0.187
Use of sofrito sauce (2 servings/week)	8 (11.9)	4 (6.3)	0.284

Values are expressed as mean ± standard deviation (SD) for quantitative variables, and as absolute and relative frequencies (%) for categorical variables.

No significant differences were detected in most of them, with the exception of the item relating to wine consumption, a finding which should be interpreted with caution. However, the overall pattern of results showed a consistent trend toward greater adherence to the MD recommendations in the IG, while in the CG the changes were more modest.

Descriptively, the intervention group showed a more favorable trend across several items of the MEDAS questionnaire. Specifically, compared with the CG, the IG showed greater increases in the use of EVOO and the consumption of vegetables, fruit and pulses, as well as a greater reduction in the consumption of commercial pastries. Improvements were also observed in the consumption of butter, margarine, cream and sugary drinks in both groups, although these were greater in the IG.

### 3.4 Changes in clinical outcomes after the intervention

Linear mixed-effects models were also fitted for systolic blood pressure, diastolic blood pressure and body mass index as secondary outcomes (Table 5). No statistically significant group x time interactions were observed for systolic blood pressures (*F* = 2.435, *p* = 0.122), diastolic blood pressure (*F* = 3.171, *p* = 0.078) or body mass index (*F* = 0.635, *p* = 0.427).

**Table 5:** Changes in secondary clinical outcomes according to study group.

Clinical outcome	CG baseline	CG 3 months	IG baseline	IG 3 months	Group × time F	*p*-value
SBP (mmHg)	129.4 ± 18.1	128.6 ± 17.0	136.2 ± 19.8	129.8 ± 16.6	2.435	0.122
DBP (mmHg)	78.7 ± 9.2	78.5 ± 9.2	82.4 ± 10.0	78.1 ± 8.6	3.171	0.078
IMC (kg/m^2^)	26.3 ± 6.5	27.0 ± 6.1	27.0 ± 5.5	26.5 ± 5.5	0.635	0.427

Values are presented as mean ± standard deviation (SD). Between-group differences in the evolution of systolic blood pressure (SBP), diastolic blood pressure (DBP), and body mass index (BMI) were evaluated using linear mixed-effects models. P values correspond to the group × time interaction.

## 4 Discussion

Overall, and despite the fact that both groups demonstrated moderate-to-high adherence to the MD prior to the intervention, the results show significant differences in the evolution of dietary habits after 3 months, with a greater increase in the number of MEDAS items met in the IG. This supports the short-term effectiveness of the multicomponent intervention in improving Mediterranean diet adherence compared with the control condition, as demonstrated by the significant adjusted group × time interaction (estimated between-group difference in change: 0.804 points; 95% CI 0.092–1.516; *p* = 0.027). These results suggest that even in populations with relatively favorable dietary patterns at baseline, structured interventions based on dietary education and practical tools can generate further improvements in diet quality. This aspect is particularly relevant, as the available evidence indicates that modest increases in adherence to the MD may be associated with clinically relevant benefits. Specifically, increases of 2–3 points in the MEDAS score have been linked to a lower incidence of cardiovascular disease, a reduced risk of cognitive decline and dementia, and a reduction in all-cause mortality (12, 34–36).

Our findings are consistent with previous studies that have shown significant improvements in MD adherence following educational or multi-component interventions in various population groups. In patients with type 2 diabetes mellitus, Cubillos et al. (37) and Gómez-Huelgas et al. (38) observed increases of around 2 points in the MEDAS score, while Alonso-Domínguez et al. (39) described an increase of 2.2 points following an intervention that combined healthy walks, a mobile app and a nutrition education workshop. Similarly, Jennings et al. (34) reported an improvement of 3.7 points at 24 weeks in individuals at risk of cognitive decline, and Recio et al. (40) observed a significant increase in adherence to the MD following a 12-month multi-level intervention. Furthermore, Cabrera-Suárez et al. (41) demonstrated favorable effects of an intervention based on online resources, the provision of EVOO and specific dietary recommendations on health-related quality of life.

In this context, the main value of our study lies in demonstrating that a brief intervention, applied to the community-dwelling elderly population and in real-life contexts of food purchasing and preparation, can improve adherence to the MD even when the baseline level of adherence is already relatively high.

### 4.1 Adherence to the MD before the procedure

Baseline scores on the MEDAS questionnaire were high in both groups (9.0 in the CG and 9.7 in the IG), indicating moderate-to-high initial adherence to the MD from the start of the study. This finding is relevant for the interpretation of subsequent results, as it suggests the possible presence of a ceiling effect, which would have limited the ability to detect further changes of greater magnitude following the intervention. In this regard, the absence of significant differences should not be interpreted as a lack of efficacy, but rather as an attenuation of the effect resulting from the characteristics of the population and the environment. The cohort studied, people aged over 60 living in Salamanca, is characterized by a dietary pattern closely aligned with the MD, notably featuring frequent consumption of fruit, EVOO, nuts and pulses, and frequent use of traditional cooking methods. This may have reduced the scope for observable improvement due to a methodological limitation arising from the cohort’s high baseline. In this context, it is plausible that interventions of this kind would have a greater impact on populations with lower baseline adherence, where there is greater scope for improvement. Nevertheless, the fact that the intervention succeeded in significantly improving the overall MEDAS score reinforces its usefulness even among older people living in the community who already have relatively favorable dietary habits.

### 4.2 Changes in dietary patterns following the intervention

The results suggest that the intervention generally led to a greater presence of foods more typical of the MD, particularly plant-based foods with cardioprotective properties. Descriptive analyses of individual MEDAS items showed a generally more favorable pattern in the intervention group, although most individual between-group differences did not reach statistical significance.

In the IG, daily consumption of vegetables and fruit increased by 25 and 12%, respectively. In Spain, although most people aged 60 and over show a higher degree of compliance with the recommended fruit intake, with Castile and León being one of the regions with the highest consumption, more than half of the population does not meet the recommendations for vegetables (13, 42–44). Various studies have shown significant increases in fruit and vegetable consumption among groups that receive intensive community-based programs combining education, practical skills and access to food (45, 46). As for legumes, despite high baseline adherence, the IG group exceeded the CG group by 7 percentage points in terms of consumption of ≥3 portions per week. This result, together with the increase in daily fruit and vegetable consumption, supports the hypothesis that although individual changes did not always reach statistical significance, the intervention implemented is associated with an overall qualitative improvement in the diet, in line with the Mediterranean model. The benefits associated with adherence to the recommendations for fruit, vegetables and pulses are attributed to the synergistic effect of dietary fiber, micronutrients and bioactive compounds present in these foods, whose nutritional profile has been linked to improvements in cardiometabolic health, mental health and healthier aging (22, 47).

Another notable aspect is the qualitative improvement in the overall lipid profile of the diet following the intervention. This change appears to be due to the increased prominence of fat sources typical of the MD, such as EVOO, nuts and fish, alongside a parallel trend toward reducing foods high in saturated fats or ultra-processed foods. The use of EVOO as the main cooking fat was already very high at the start of the study (>90% in both groups), which limited the scope for improvement. However, an increase in compliance with the recommendation of ≥4 tablespoons/day was observed, particularly in the IG group. This finding reinforces adherence to current recommendations, which promote the regular use of EVOO not only as a substitute for saturated fats, but as a central component of the MD, associated with the prevention of chronic diseases and improved quality of life (48, 49). A daily intake of between 20 and 40 grams is recommended, based on the findings of numerous studies, notably the PREDIMED study (17), which found a reduction in the incidence of type 2 diabetes in more than half of the participants who supplemented their diet with EVOO or nuts (17).

Regarding weekly nut consumption, although the increase was greater in the CG, this result must be interpreted considering baseline differences, as the IG started from higher levels of compliance. This food, rich in unsaturated fatty acids and bioactive compounds, has been widely linked to cardiovascular benefits, as well as positive effects on cognitive and psychosocial health and quality of life (35, 47). On the other hand, both groups showed a similar increase in the proportion of participants who consumed ≥3 portions of fish or seafood per week. This finding is significant from a qualitative perspective, as these foods are a major source of n-3 polyunsaturated fatty acids, which are associated with cardiometabolic, cognitive and functional benefits in older adults (35, 47, 50, 51).

At the same time, the intervention also appeared to encourage a reduction in the consumption of less healthy foods or those consumed only occasionally. Compared with the CG, the IG showed a more favorable trend in the reduction of butter, margarine, cream, sugary drinks and commercial pastries. This finding suggests that the intervention not only improved the inclusion of protective foods, but also the ability to replace ultra-processed products or those high in saturated fats and sugars with options more in line with the MD. In this regard, our results are consistent with previous studies in which educational interventions offering practical support with planning, shopping and reading labels were associated with a reduction in the consumption of less healthy products (52).

With regard to the consumption of red and processed meat, although the CG had a higher proportion of participants who reduced their intake to recommended levels, this result should be interpreted with caution due to baseline differences between the groups; indeed, 20% more participants in the IG were already complying with the established recommendation compared with the CG before the start of the study. The importance of reducing the consumption of red and processed meat is reflected in most current dietary guidelines, which recommend limited consumption of red and processed meat (42, 53, 54). Similarly, the Spanish Agency for Food Safety and Nutrition (54) recommends limiting consumption to two portions or fewer per week. It is worth noting that changes in red meat consumption may be influenced by socio-cultural and economic factors, such as a strong tradition of livestock farming and the importance of the meat sector in certain regions. In this context, Castile and León tops the list of regions with the highest consumption of red meat, particularly among those aged 65 and over, exceeding the national average. This reveals a deeply rooted gastronomic culture in this geographical area that may hinder significant changes in the short term (15, 44).

About wine consumption, a significant 10% difference in the IG was observed compared with the CG. However, this item has been considered a variable of complex interpretation and, therefore, this data should be interpreted with caution, as although some observational studies have described associations between low alcohol consumption and certain favorable cardiovascular outcomes, the current evidence is controversial and does not justify recommending the initiation or increase of wine consumption as a health strategy. In fact, the main clinical guidelines, including those of the American Heart Association (55), the SEOM (56) and the European Code Against Cancer (57), state that people who do not consume alcohol should not start doing so for potential cardiovascular benefits, and that higher levels of consumption are consistently associated with worse cardiovascular outcomes. They also emphasize that there is no safe level of alcohol consumption in relation to cancer risk. Therefore, in the present study, this variable is not considered a key indicator of improvement, but rather a secondary finding whose interpretation remains controversial (55–57).

Although the intervention significantly improved adherence to the Mediterranean diet, no significant changes were observed in body mass index or blood pressure. This finding is likely explained by the relatively short follow-up period, during which improvements in dietary behavior may not yet translate into measurable anthropometric or cardiovascular changes.

### 4.3 Probable mechanisms underlying the effectiveness of the multicomponent intervention

The observed effectiveness of the intervention likely cannot be attributed to a single component, but rather to the synergistic effect of combining multiple behavior change strategies. Nutrition education may have improved participants’ knowledge of the principles of the Mediterranean diet, while practical workshops provided opportunities to translate this knowledge into everyday skills through experiential learning, including selecting healthier foods and preparing meals. Furthermore, the group format may have enhanced social support, peer learning, and motivation, while periodic reinforcement of educational messages throughout the intervention may have contributed to increasing participants’ self-efficacy and facilitating the consolidation of healthier eating habits. These findings are consistent with recent evidence showing that Mediterranean diet interventions for older adults are more effective when they integrate complementary behavior change techniques, particularly hands-on instruction, experiential learning, social support, and guidance from credible health professionals, rather than relying solely on nutrition education (58). Furthermore, theories of behavior change, particularly those based on social cognitive theory, identify self-efficacy as a central mechanism for achieving and maintaining long-term dietary behavior change (59). Although the present study was not designed to specifically assess these mechanisms, it is likely that the combination of these factors explains, at least in part, the observed improvement in adherence to the Mediterranean diet.

### 4.4 The potentials clinical and public health implications of these changes

The findings of this study are of particular clinical and public health relevance, as they show that a multi-component intervention based on nutritional literacy and practical skills can improve adherence to MD among older people living in the community, even in the absence of a specific medical condition acting as a motivating factor for change.

About interventions targeting the older population, most of the studies identified were designed for adults with a pre-existing condition, which could skew the results toward greater adherence, as the health belief model demonstrates that people with a diagnosed condition tend to show greater motivation to modify their eating behavior compared to healthy individuals. Furthermore, much of the existing literature provides participants in nutritional interventions with foods typical of MD free of charge or financial incentives in the form of vouchers, which makes their consumption more likely and, consequently, increases the observed adherence (2, 3, 10–12, 17, 47, 52, 60). In contrast, there are still very few programs specifically aimed at people over the age of 60 that are based on practical nutrition education, cooking workshops and training in how to read nutrition labels.

### 4.5 Limitations of the study

This study has several limitations that should be considered when interpreting the findings. First, the follow-up period was limited to 3 months, precluding conclusions regarding the long-term maintenance of the observed improvements in Mediterranean diet adherence. As dietary behavior change is a dynamic process that requires continued reinforcement, further studies with longer follow-up are needed to determine the durability of these effects and their potential implications for long-term health outcomes.

Second, the study population consisted of community-dwelling older adults participating in a municipal program in Salamanca and was predominantly female (83%). Although an exploratory analysis did not identify significant sex-related differences in the intervention effect, the relatively small number of male participants limited the statistical power to detect potential sex-specific differences. Consequently, the findings should be interpreted with caution and may not be fully generalizable to older men or to other populations or settings. In addition, participants showed relatively high baseline adherence to the Mediterranean diet, which may have limited the potential for improvement and attenuated the magnitude of the intervention effect. Consequently, caution is warranted when generalizing these findings to other populations or settings.

Another limitation relates to the assessment of Mediterranean diet adherence using the Mediterranean Diet Adherence Screener (MEDAS). Although MEDAS is a validated and widely used instrument specifically designed to assess adherence to the Mediterranean dietary pattern, it is a self-reported measure that provides only a brief assessment of dietary habits and may therefore be subject to recall and social desirability biases. Furthermore, it does not provide the detailed dietary intake information obtained from food frequency questionnaires or repeated dietary recalls, nor the objective assessment afforded by nutritional biomarkers. Nevertheless, because the primary objective of this trial was to evaluate changes in adherence to the Mediterranean dietary pattern rather than to quantify dietary intake, MEDAS was considered the most appropriate primary outcome measure. More comprehensive dietary assessment methods would therefore have complemented, rather than replaced, the primary outcome selected for this study.

Finally, the assumptions used for the sample size calculation were during the study design based on the best evidence available at the time, as no directly comparable intervention studies were available to inform these parameters. Although this approach was considered appropriate during the planning phase, these assumptions should be interpreted in the context of the available evidence at the time study was designed.

These limitations should be considered when interpreting the findings; however, they do not detract from the internal validity of the study, and the randomized controlled design strengthens the reliability of the observed intervention effects.

## 5 Conclusion

The multicomponent intervention aimed was more effective than a traditional group educational intervention in improving adherence to the Mediterranean diet in community-dwelling older adults, as demonstrated by the significantly greater improvement in MEDAS scores over time. Descriptive analyses also suggested favorable changes across most MEDAS items, particularly in the consumption of olive oil, fruit, vegetables and legumes, together with a reduction in less healthy food choices. These improvements were still evident at the three-month follow-up assessment.

Overall, these findings support the feasibility and short-term effectiveness of multicomponent behavior-change interventions combining nutrition education with practical food purchasing and cooking workshops to improve adherence to the Mediterranean diet in older adults. By promoting nutritional literacy, practical food-related skills, autonomy in food choices, and social interaction, these interventions may represent a feasible strategy for community-based health promotion programs. Nevertheless, further studies with longer follow-up are required to determine whether these behavioral changes can be maintained over time and translated into long-term health benefits at the population level.

## Data Availability

The original contributions presented in the study are included in the article/Supplementary material, further inquiries can be directed to the corresponding author.

## Supplementary material

SUPPLEMENTARY FIGURE S1 Educational materials promoting healthy eating and lifestyle habits among older adults.

SUPPLEMENTARY FIGURE S2 Educational materials used in the shopping basket workshop to improve nutrition literacy and healthy food purchasing behaviors among older adults.

## References

[1] Suárez-Moreno N Gómez-Sánchez L Navarro-Caceres A Arroyo-Romero S Domínguez-Martín A Lugones-Sánchez C . Association of Mediterranean diet with cardiovascular risk factors and with metabolic syndrome in subjects with long COVID: BioICOPER study. Nutrients. (2025) 17:656. doi: 10.3390/nu17040656, 40004984 PMC11858499

[2] Ecarnot F Maggi S. The impact of the Mediterranean diet on immune function in older adults. Aging Clin Exp Res. (2024) 36:117. doi: 10.1007/s40520-024-02753-3, 38780713 PMC11116168

[3] Hu FB. Diet strategies for promoting healthy aging and longevity: an epidemiological perspective. J Intern Med. (2024) 295:508–31. doi: 10.1111/joim.13728, 37867396 PMC10939982

[4] Godos J Guglielmetti M Ferraris C Frias-Toral E Domínguez Azpíroz I Lipari V . Mediterranean diet and quality of life in adults: a systematic review. Nutrients. (2025) 17:577. doi: 10.3390/nu17030577, 39940436 PMC11819740

[5] Ni J Hernández-Cacho A Nishi SK Babio N Belzer C Konstati P . Mediterranean diet, gut microbiota, and cognitive decline in older adults with obesity/overweight and metabolic syndrome: a prospective cohort study. BMC Med. (2025) 23:669. doi: 10.1186/s12916-025-04488-y, 41327300 PMC12667113

[6] Pérez-Ros P Rios-Corral A Cauli O. Association between Mediterranean diet consumption and the physical and mental components of HRQL in community-dwelling older adults in Valencia. Nutrients. (2025) 17:3243. doi: 10.3390/nu17203243, 41156496 PMC12567089

[7] Srambikkal Vinesh D Syed Altaf RR Mohan A Palani N Mendonce KC Selvan S . The Mediterranean diet as a metabolic strategy for healthy aging and non-communicable disease prevention. J Nutr Physiol. (2026) 5:100015. doi: 10.1016/j.jnphys.2026.100015

[8] Tessier AJ Wang F Korat AA Eliassen AH Chavarro J Grodstein F . Optimal dietary patterns for healthy aging. Nat Med. (2025) 31:1644–52. doi: 10.1038/s41591-025-03570-5, 40128348 PMC12092270

[9] Instituto Nacional de Estadística. (2024). Proporción de Personas Mayores de Cierta edad por provincia. Available online at: https://www.ine.es/jaxiT3/Datos.htm?t=1488

[10] Conde-Pipó J Martinez-Amat A Mora-Fernández A Mariscal-Arcas M. Impact of Mediterranean diet pattern adherence on the physical component of health-related quality of life in middle-aged and older active adults. Nutrients. (2024) 16:3877. doi: 10.3390/nu16223877, 39599663 PMC11597341

[11] Hidalgo-Liberona N Meroño T Zamora-Ros R Rabassa M Semba R Tanaka T . Adherence to the Mediterranean diet assessed by a novel dietary biomarker score and mortality in older adults: the InCHIANTI cohort study. BMC Med. (2021) 19:280. doi: 10.1186/s12916-021-02154-7, 34814922 PMC8611910

[12] Shannon OM Ranson JM Gregory S Macpherson H Milte C Lentjes M . Mediterranean diet adherence is associated with lower dementia risk, independent of genetic predisposition: findings from the UK biobank prospective cohort study. BMC Med. (2023) 21:81. doi: 10.1186/s12916-023-02772-3, 36915130 PMC10012551

[13] Martínez Valero AP Amo-Saus E Pardo-García I Escribano-Sotos F. Diet quality in a population aged over 65 and related socioeconomic factors. Aten Primaria. (2021) 53:27–35. doi: 10.1016/j.aprim.2019.12.001, 32143973 PMC7752958

[14] Navarro-Martínez R Mafla-España MA Cauli O. Mediterranean diet adherence in community-dwelling older adults in Spain: social determinants related to the family. Nutrients. (2022) 14:5141. doi: 10.3390/nu14235141, 36501170 PMC9736247

[15] Ministerio de Sanidad. Encuesta Europea de Salud en España 2020 (EESE 2020) (2020).

[16] Guías Alimentarias de la Sociedad Española de Nutrición Comunitaria (SENC) para la Población Española Guías Alimentarias de la Sociedad Española de Nutrición Comunitaria (SENC) para la Población Española; las nuevas Pirámides (SENC, 2025/26) de la Alimentación, la Gastronomía y la Hidratación Saludables y Sostenibles*. Dietary Guidelines of the Spanish Society of Community Nutrition (SENC) for the Spanish Population; the new Pyramids (SENC, 2025/26) for Healthy and Sustainable Food, Gastronomy, and Hydration Summary. Madrid, Spain: Editorial Médica Panamericana.

[17] Salas-Salvadó J Bulló M Babio N Martínez-González MÁ Ibarrola-Jurado N Basora J . Reduction in the incidence of type 2 diabetes with the Mediterranean diet: results of the PREDIMED-Reus nutrition intervention randomized trial. Diabetes Care. (2011) 34:14–9. doi: 10.2337/dc10-1288, 20929998 PMC3005482

[18] Konieczna J Ruiz-Canela M Galmes-Panades AM Abete I Babio N Fiol M . An energy-reduced Mediterranean diet, physical activity, and body composition: an interim subgroup analysis of the PREDIMED-plus randomized clinical trial. JAMA Netw Open. (2023) 6:e2337994. doi: 10.1001/jamanetworkopen.2023.37994, 37851444 PMC10585413

[19] Uzdil Z Çelebi MSM Öztürk YE Kaya PS. Effect of food literacy on adherence to Mediterranean diet and anthropometric measurements in adults. Rev Nutr. (2024) 37:e230034. doi: 10.1590/1678-9865202437e230034

[20] Öngün Yilmaz H Arslan S Tari Selçuk K Bayram HM Ozturkcan A. From knowledge to action: how nutrition knowledge shapes sustainable eating and Mediterranean diet adherence. Front Nutr. (2025) 12:1684438. doi: 10.3389/fnut.2025.1684438, 41334339 PMC12665548

[21] Schröder H Fitó M Estruch R Martínez-González MA Corella D Salas-Salvadó J . A short screener is valid for assessing Mediterranean diet adherence among older Spanish men and women. J Nutr. (2011) 141:1140–5. doi: 10.3945/jn.110.135566, 21508208

[22] Maderuelo-Fernandez JA Recio-Rodríguez JI Patino-Alonso MC Pérez-Arechaederra D Rodriguez-Sanchez E Gomez-Marcos MA . Effectiveness of interventions applicable to primary health care settings to promote Mediterranean diet or healthy eating adherence in adults: a systematic review. Prev Med. (2015) 76:S39–55. doi: 10.1016/j.ypmed.2014.12.011, 25524613

[23] Navarrete-Muñoz EM Torres-Collado L Valera-Gran D Gonzalez-Palacios S Compañ-Gabucio LM Hernández-Sánchez S . Nutrition labelling use and higher adherence to Mediterranean diet: results from the DiSA-UMH study. Nutrients. (2018) 10:442. doi: 10.3390/nu10040442, 29614009 PMC5946227

[24] Pfeifer D Rešetar J Gajdoš Kljusurić J Panjkota Krbavčić I Vranešić Bender D Rodríguez-Pérez C . Cooking at home and adherence to the Mediterranean diet during the COVID-19 confinement: the experience from the Croatian COVIDiet study. Front Nutr. (2021) 8:617721. doi: 10.3389/fnut.2021.617721, 33869262 PMC8044460

[25] da Fonseca PG Siqueira L d C Raposo A Alslamah T Albaridi NA Saraiva A . From kitchen to health: how culinary workshops influence eating habits, autonomy, and wellbeing in adults–a scoping review. Front Nutr. (2025) 12:1653406. doi: 10.3389/fnut.2025.1653406 PMC12425794 40948859

[26] Casucci MG Muñoz-Martínez J Caneda-Ferrón B Salinas-Roca B Orta-Ramirez A Vidal E . The association between Mediterranean diet -related health literacy, cooking skills and Mediterranean diet adherence in the Spanish population. Nutrients. (2026) 18:235. doi: 10.3390/nu18020235, 41599849 PMC12845093

[27] Lorca-Camara V Bach-Faig A Bes-Rastrollo M Jurado-Gonzalez P O’Callaghan-Gordo C Bosque-Prous M. Adherence to the Mediterranean diet and its association with sustainable eating knowledge, attitudes, habits, and cooking self-efficacy among Spanish adults: a cross-sectional study. Sustainability. (2025) 17:8580. doi: 10.3390/su17198580

[28] De Marco S Marziali E Nachira L Arcaro P Villani L Galasso V . A multidisciplinary primary prevention intervention to increase adherence to the Mediterranean diet: a pilot study. BMC Public Health. (2023) 23:2051. doi: 10.1186/s12889-023-16893-0, 37980473 PMC10657599

[29] Sánchez González JL Pérez Robledo F Bermejo Gil BM Domínguez García A Artigas Martín JE Sánchez Gómez C Evaluación, indicación y seguimiento del programa de actividad física en personas mayores y realización de programas de revitalización y terapia ocupacional. Programa de Revitalización Geriátrica Asociación Universitaria de Educación y Psicología (ASUNIVEP). Almería, Spain: Asociación Universitaria de Educación y Psicología (ASUNIVEP) (2018).

[30] Fernández-Ballart JD Piñol JL Zazpe I Corella D Carrasco P Toledo E . Relative validity of a semi-quantitative food-frequency questionnaire in an elderly Mediterranean population of Spain. Br J Nutr. (2010) 103:1808–16. doi: 10.1017/S0007114509993837, 20102675

[31] Papadaki A Nolen-Doerr E Mantzoros CS. The effect of the mediterranean diet on metabolic health: a systematic review and meta-analysis of controlled trials in adults. Nutrients. (2020) 12:3342. doi: 10.3390/nu12113342, 33143083 PMC7692768

[32] Stergiou GS Palatini P Parati G O’Brien E Januszewicz A Lurbe E . 2021 European Society of Hypertension practice guidelines for office and out-of-office blood pressure measurement. J Hypertens. (2021) 39:1293–302. doi: 10.1097/HJH.0000000000002843, 33710173

[33] World Medical Association. World medical association declaration of Helsinki. JAMA. (2025) 333:71. doi: 10.1001/jama.2024.21972, 39425955

[34] Jennings A Shannon OM Gillings R Lee V Elsworthy R Bundy R . Effectiveness and feasibility of a theory-informed intervention to improve Mediterranean diet adherence, physical activity and cognition in older adults at risk of dementia: the MedEx-UK randomised controlled trial. BMC Med. (2024) 22:600. doi: 10.1186/s12916-024-03815-z, 39716203 PMC11667912

[35] Estruch R Ros E Salas-Salvadó J Covas MI Corella D Arós F . Primary prevention of cardiovascular disease with a Mediterranean diet supplemented with extra-virgin olive oil or nuts. N Engl J Med. (2018) 378:e34. doi: 10.1056/nejmoa1800389, 29897866

[36] Furbatto M Lelli D Antonelli Incalzi R Pedone C. Mediterranean diet in older adults: cardiovascular outcomes and mortality from observational and interventional studies—a systematic review and meta-analysis. Nutrients. (2024) 16:3947. doi: 10.3390/nu16223947, 39599734 PMC11597443

[37] Cubillos L Estrada del Campo Y Harbi K Keyserling T Samuel-Hodge C Reuland DS. Feasibility and acceptability of a clinic-based Mediterranean-style diet intervention to reduce cardiovascular risk for Hispanic Americans with type 2 diabetes. Diabetes Educator. (2017) 43:286–96. doi: 10.1177/0145721717706030, 28427311 PMC5800306

[38] Gomez-Huelgas R Jansen-Chaparro S Baca-Osorio AJ Mancera-Romero J Tinahones FJ Bernal-López MR. Effects of a long-term lifestyle intervention program with Mediterranean diet and exercise for the management of patients with metabolic syndrome in a primary care setting. Eur J Intern Med. (2015) 26:317–23. doi: 10.1016/j.ejim.2015.04.007, 25907985

[39] Alonso-Domínguez R García-Ortiz L Patino-Alonso MC Sánchez-Aguadero N Gómez-Marcos MA Recio-Rodríguez JI. Effectiveness of a multifactorial intervention in increasing adherence to the Mediterranean diet among patients with diabetes mellitus type 2: a controlled and randomized study (EMID study). Nutrients. (2019) 11:162. doi: 10.3390/nu11010162, 30646500 PMC6357113

[40] Recio-Rodriguez JI Garcia-Ortiz L. Garcia-Yu IA Lugones-Sanchez C. Zabaleta-del-Olmo E Bolibar B. Effectiveness of a multiple health-behaviour-change intervention in increasing adherence to the Mediterranean diet in adults (EIRA study): a randomized controlled hybrid trial. BMC Public Health (2022);22:2127. doi: 10.1186/s12889-022-14590-y PMC9675247 36401247

[41] Cabrera-Suárez BM Lahortiga-Ramos F Sayon-Orea C Hernández-Fleta JL González-Pinto A Molero P . Effect of a dietary intervention based on the Mediterranean diet on the quality of life of patients recovered from depression: analysis of the PREDIDEP randomized trial. Exp Gerontol. (2023) 175:112149. doi: 10.1016/j.exger.2023.112149, 36933773

[42] EFSA Panel on Dietetic Products, Nutrition, and Allergies (NDA). Scientific opinion on establishing food-based dietary guidelines. EFSA J. (2010) 8:1460. doi: 10.2903/j.efsa.2010.1460

[43] EFSA Panel on Dietetic Products, Nutrition, and Allergies (NDA). Scientific opinion on dietary reference values for carbohydrates and dietary fibre. EFSA J. (2010) 8:1462. doi: 10.2903/j.efsa.2010.1462

[44] Ministerio de Agricultura, Pesca y Alimentación. Informe del consumo alimentario en España 2024. Madrid: Ministerio de Agricultura, Pesca y Alimentación, Secretaría General Técnica, Centro de Publicaciones (2025). https://servicio.mapa.gob.es/tienda/.

[45] Smith E Sutarso T Kaye GL. Access with education improves fruit and vegetable intake in preschool children. J Nutr Educ Behav. (2020) 52:145–51. doi: 10.1016/j.jneb.2019.07.016, 31494058

[46] Metcalfe JJ Prescott MP Schumacher M Kownacki C McCaffrey J. Community-based culinary and nutrition education intervention promotes fruit and vegetable consumption. Public Health Nutr. (2022) 25:437–449. doi: 10.1017/S1368980021003797, 34482851 PMC8883782

[47] Parletta N Zarnowiecki D Cho J Wilson A Bogomolova S Villani A . A Mediterranean-style dietary intervention supplemented with fish oil improves diet quality and mental health in people with depression: a randomized controlled trial (HELFIMED). Nutr Neurosci. (2019) 22:474–87. doi: 10.1080/1028415X.2017.1411320, 29215971

[48] Aranceta Bartrina J. Guías alimentarias para la población española (SENC, 2016); la nueva pirámide de la alimentación saludable. Nutr Hosp. (2016) 33:1–48. doi: 10.20960/nh.827 28196425

[49] Rubio López JM. Oil and life: the impact of olive oil on human health. A review of the evidence supporting the recommendation for extra virgin olive oil consumption. Semergen. (2025) 51:102582. doi: 10.1016/j.semerg.2025.102582, 40929788

[50] Foscolou A Pratti T Detopoulou P Argyri K Nikolaou G Kouskouti A . Dietary n-3 polyunsaturated fatty acids from fish are associated with better healthy aging indicators: results of the DIAPELH study. J Hum Nutr Diet. (2025) 38:e70169. doi: 10.1111/jhn.70169, 41277355 PMC12641236

[51] Chen Y Yang L Wang K An Y Wang Y Zheng Y . Relationship between fatty acid intake and aging: a Mendelian randomization study. Aging. (2024) 16:5711–39. doi: 10.18632/aging.205674 PMC11006485 38535988

[52] Mayr HL Tierney AC Kucianski T Thomas CJ Itsiopoulos C. Australian patients with coronary heart disease achieve high adherence to 6-month Mediterranean diet intervention: preliminary results of the AUSMED heart trial. Nutrition. (2019) 61:21–31. doi: 10.1016/j.nut.2018.10.027, 30682704

[53] Arnett DK Blumenthal RS Albert MA Buroker AB Goldberger ZD Hahn EJ . ACC/AHA guideline on the primary prevention of cardiovascular disease: a report of the American College of Cardiology/American Heart Association task force on clinical practice guidelines. Circulation. (2019) 140:e596–646. doi: 10.1161/CIR.0000000000000678, 30879355 PMC7734661

[54] Martínez Hernández JA Cámara Hurtado M Maria Giner Pons R González Fandos E López García E Mañes Vinuesa J Informe del Comité Científico de la Agencia Española de Seguridad Alimentaria y Nutrición (AESAN) de revisión y actualización de las Recomendaciones Dietéticas para la población española. Madrid, Spain: Agencia Española de Seguridad Alimentaria y Nutrición (AESAN) (2020).

[55] Piano MR Marcus GM Aycock DM Buckman J Hwang CL Larsson SC . Alcohol use and cardiovascular disease: a scientific statement from the American Heart Association. Circulation. (2025) 152:e7–21. doi: 10.1161/CIR.0000000000001341, 40485439

[56] Sociedad Española de Oncología Médica (SEOM) Las Cifras del Cáncer en España 2026 (2025).

[57] International Agency for Research on Cancer (IARC) Alcohol: European Code against Cancer. 5th ed IARC. (2025) Available online at: https://www.who.int/

[58] Turner A LaMonica HM Flood VM. Behaviour change techniques used in Mediterranean diet interventions for older adults: a systematic scoping review. Nutrients. (2023) 15:1189. doi: 10.3390/nu15051189, 36904188 PMC10005068

[59] Zaslavsky O Su Y Kim B Roopsawang I Wu KC Renn BN. Behavior change factors and retention in dietary interventions for older adults: a scoping review. Gerontologist. (2022) 62:e534–54. doi: 10.1093/geront/gnab133, 34477843 PMC9756309

[60] Duarte Junior MA Cabanas-Sánchez V Pintos-Carrillo S Ortolá R Rodríguez-Artalejo F Sotos-Prieto M . Association of adherence to Mediterranean diet and changes over time with all-cause mortality in older adults: the seniors-ENRICA cohorts. Am J Clin Nutr. (2025) 122:255–62. doi: 10.1016/j.ajcnut.2025.04.027, 40294749

