# Correction: Effect of a multicomponent intervention on adherence to the Mediterranean diet in older adults: a randomized controlled trial

**DOI:** 10.3389/fnut.2026.1979171

**Published:** 2026-09-08

**Authors:** Ana María Murillo-Zaldívar, Rosario Alonso-Domínguez, Jesús González-Sánchez, Teresa Vicente-García, José Ignacio Recio-Rodríguez, Fausto José Barbero-Iglesias, Maria Luz Sánchez-Tocino, Laura García-Barrueco, Maria García-Oterino, Susana González-Manzano

**Affiliations:** 1 Complejo Asistencial Universitario de Salamanca, Instituto de Investigación Biomédica de Salamanca (IBSAL), Salamanca, Spain; 2 Departamento de Enfermería y Fisioterapia, Facultad de Enfermería y Fisioterapia, Instituto de Investigación Biomédica de Salamanca, Atención de Enfermería y Fisioterapia en promoción de la salud, estilos de vida y discapacidad, Universidad de Salamanca, Salamanca, Spain; 3 Gerencia de Atención Primaria de Salamanca, Salamanca, Spain; 4 Grupo de Investigación en polifenoles, Departamento de Química Analítica, Nutrición y Bromatología, Facultad de Farmacia, Instituto de Investigación Biomédica de Salamanca, Universidad de Salamanca, Salamanca, Spain

**Keywords:** diet, elderly nutrition, food and nutrition education, food labeling, healthy, Mediterranean diet, nutrition programs, older adults

The figure captions were in the wrong order in the published version of this paper. The caption of **Figure 1** was incorrectly assigned to **Figure 2**, and the caption of **Figure 2** was incorrectly assigned to **Figure 1**.

The correct figure captions are:

**Figure 1**. Study flowchart: participant enrolment and study completion.

**Figure 2**. Estimated marginal means of MEDAS score according to study group obtained from the unadjusted linear mixed-effects models.

The original version of this article has been updated.

