# Supplementary material for: Effect of a multicomponent intervention on adherence to the Mediterranean diet in older adults: a randomized controlled trial
Source: Front Nutr. 2026 Aug 20;13:1918299. doi: 10.3389/fnut.2026.1918299 (PMC13537966; doi:10.3389/fnut.2026.1918299)

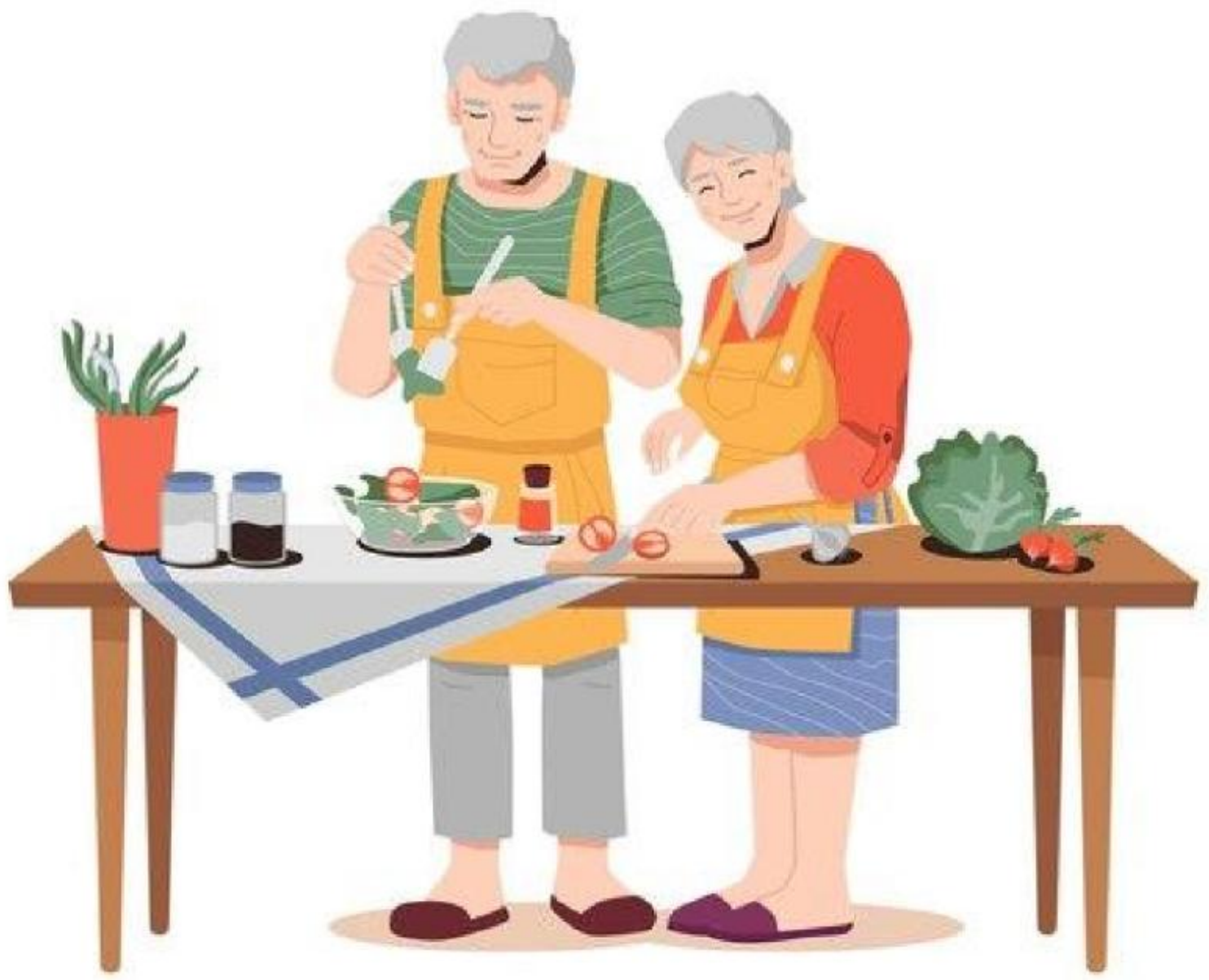

HEALTHY EATING FOR OLDER PEOPLE

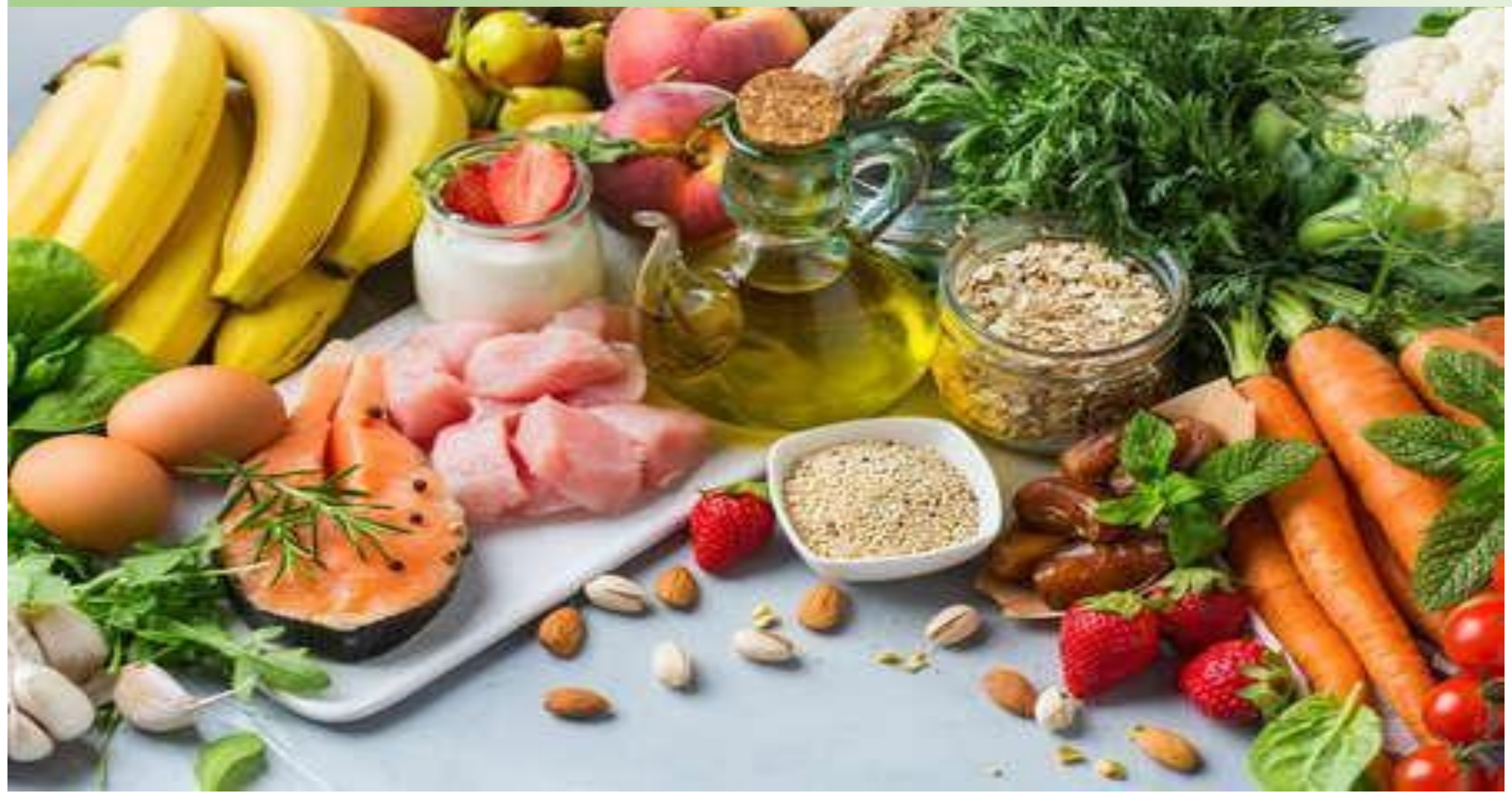

Small changes

TO EAT BETTER

More	Change to	Less
FRUITS AND VEGETABLE	WATER	SALT
LEGUMES	WHOLE FOODS	SUGARS
NUTS	VIRGIN OLIVE OIL	RED AND PROCESSED MEAT
ACTIVE AND SOCIAL LIFE	SEASONAL AND LOCAL FOODS	ULTRA-PROCESSED FOOD

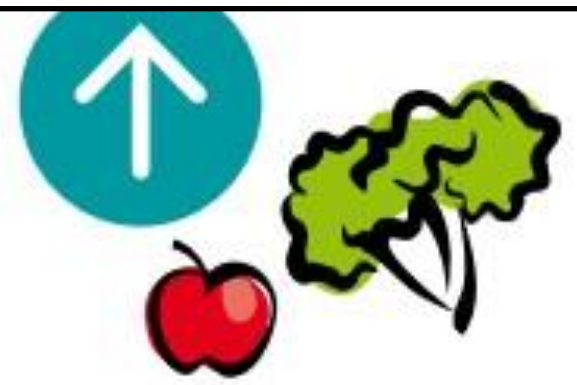

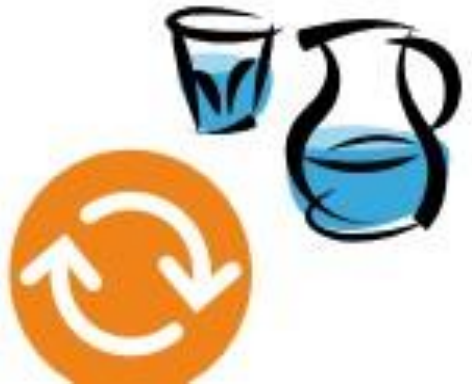

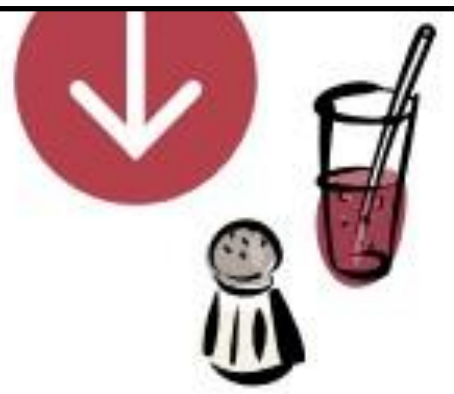

¡REMEMBER!

FRUIT AND A DAILY ROUTINE FOR LIVING TO BE A CENTENARIAN

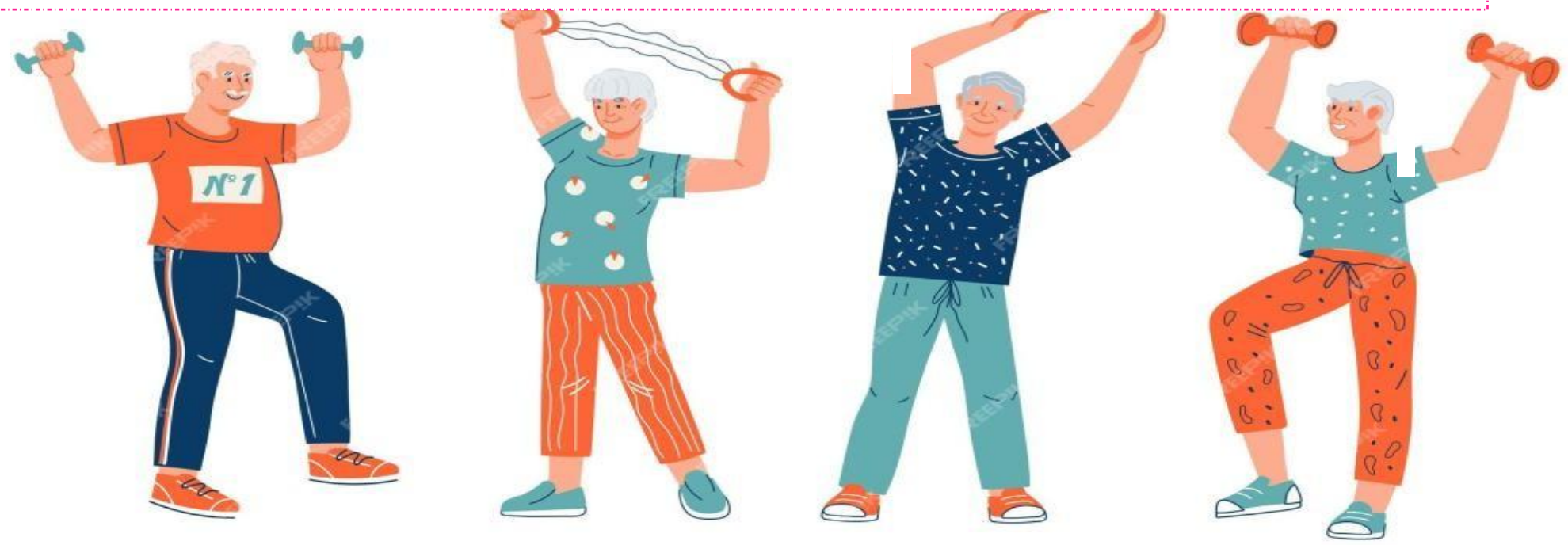

VNiVERSiDAD D SALAMANCA

Ana Mª Murillo Zaldívar

Ayuntamiento de Salamanca

OPTIONAL, OCCASIONAL AND MODERATE CONSUMPTION

Spreadable fats, sweets and industrial baked goods

Red meat, processed meat or sausages

VARIED AND DAILY CONSUMPTION

- Dairy products: 2–3 a day
- Fish, lean meats, white meats, pulses, nuts and eggs: 1–3 portions a day (vary your choices).

DAILY INTAKE AT EACH MAIN MEAL

- Vegetables: 2–3 portions a day.
- Fruit: 3–4 portions a day
- Extra virgin olive oil

- Wholemeal bread, wholemeal pasta and brown rice, potatoes, chestnuts, and tender pulses.

Depending on the level of physical activity

MAINTAINING HEALTHY LIFESTYLE

- Daily physical activity, emotional well-being, energy balance, healthy cooking techniques.
- Water: 4–6 glasses a day.

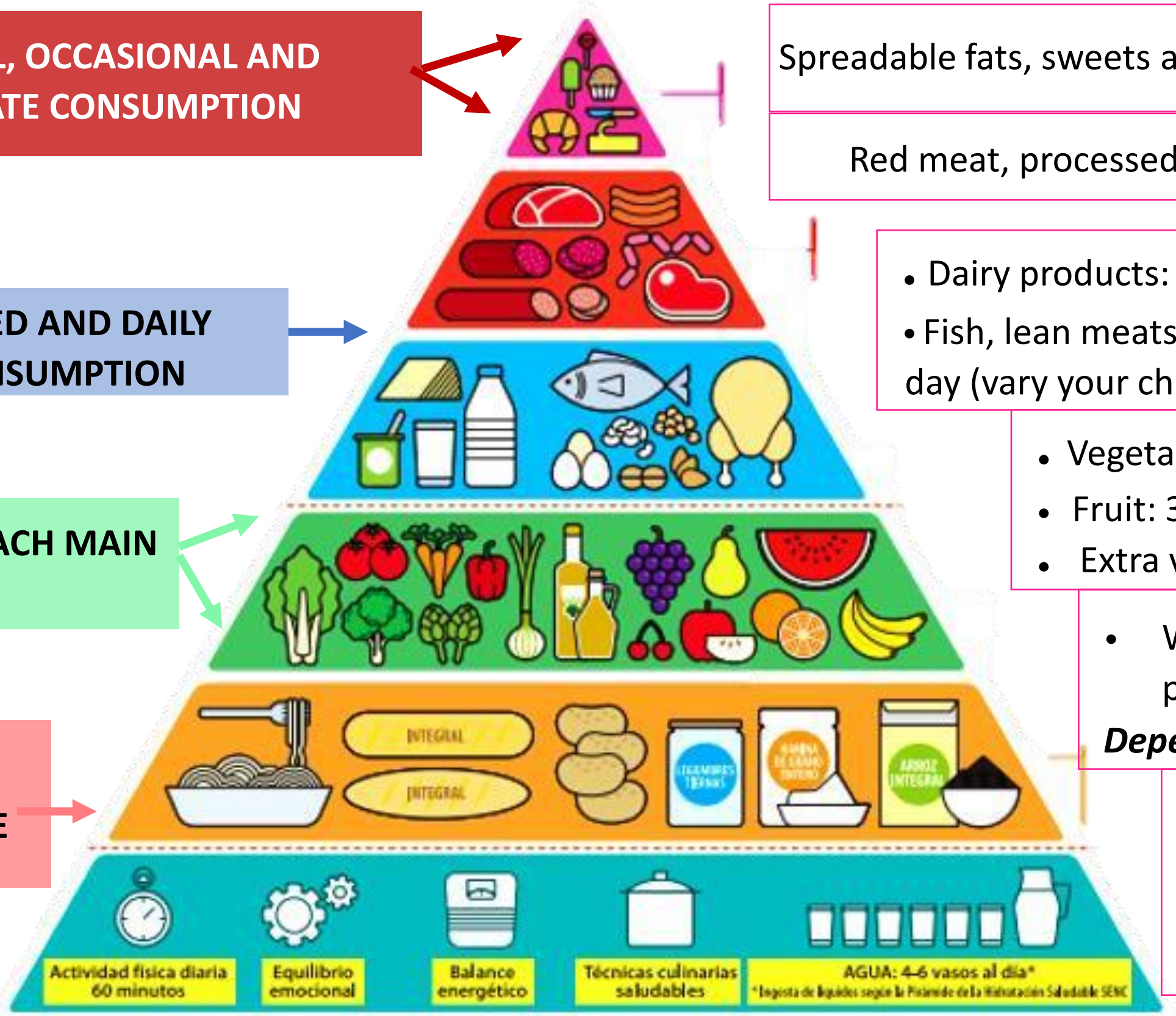

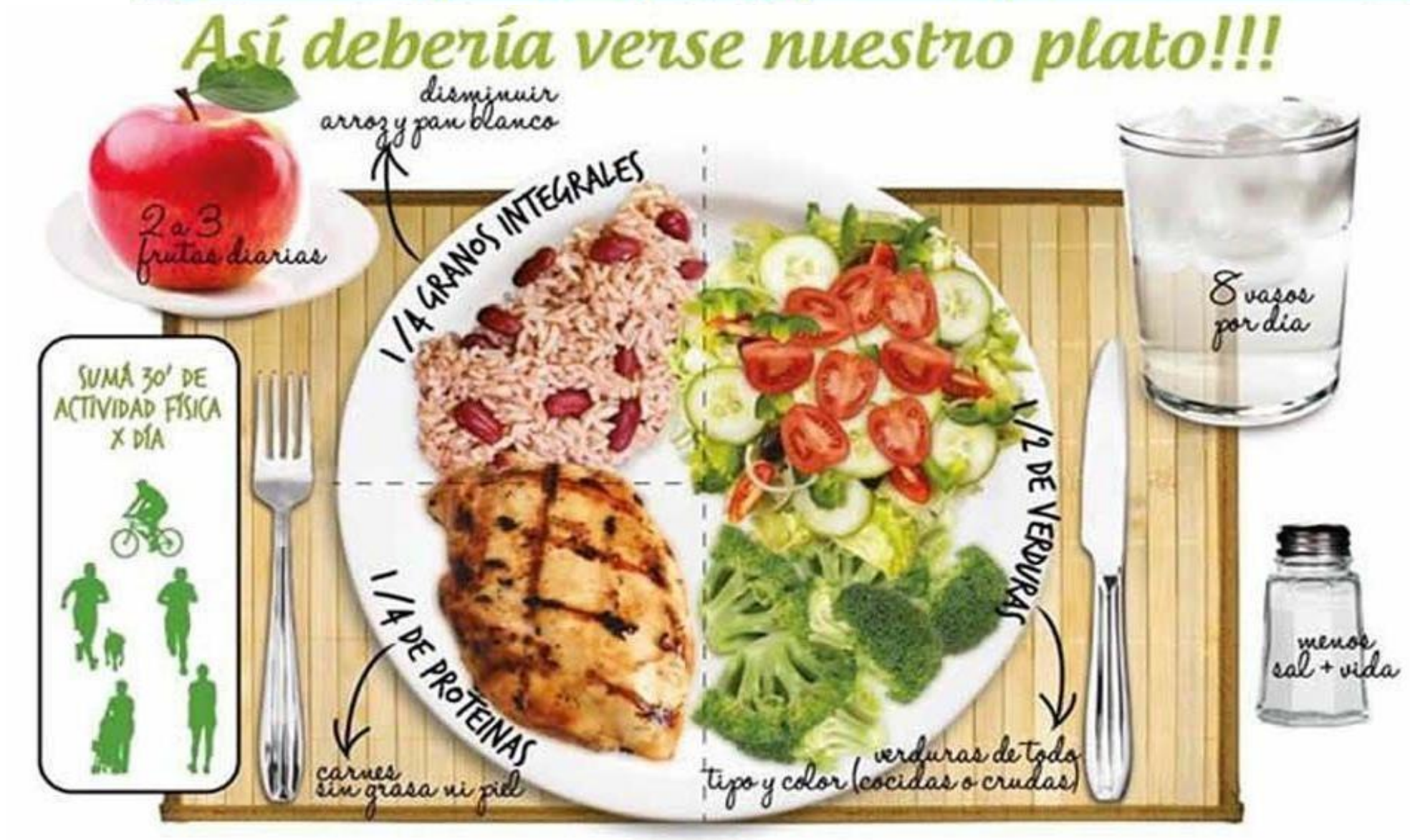

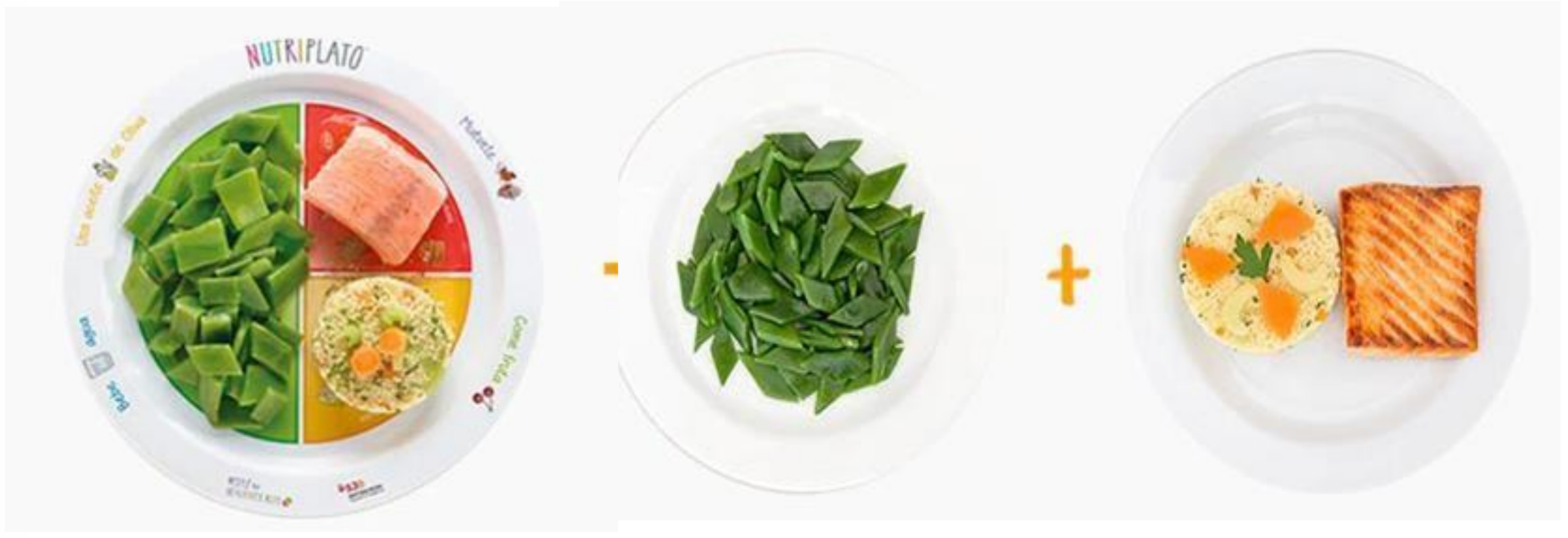

Supplement: Supplementary file 1 [file Data_Sheet_1.pdf]
