# Supplementary material for: Effect of a multicomponent intervention on adherence to the Mediterranean diet in older adults: a randomized controlled trial
Source: Front Nutr. 2026 Aug 20;13:1918299. doi: 10.3389/fnut.2026.1918299 (PMC13537966; doi:10.3389/fnut.2026.1918299)

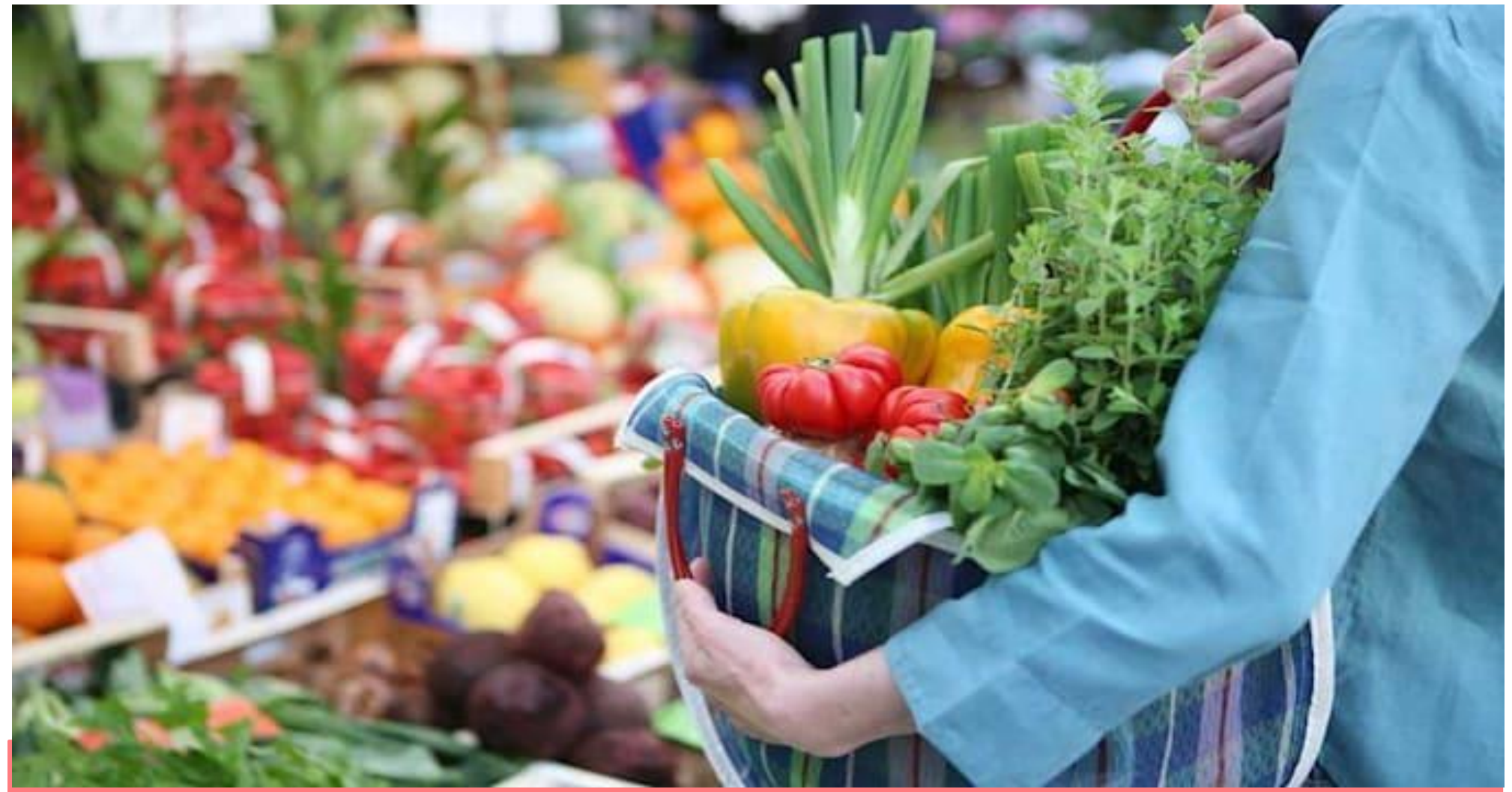

SHOPPING BASKET WORKSHOP
“VENREDI PROJECT”

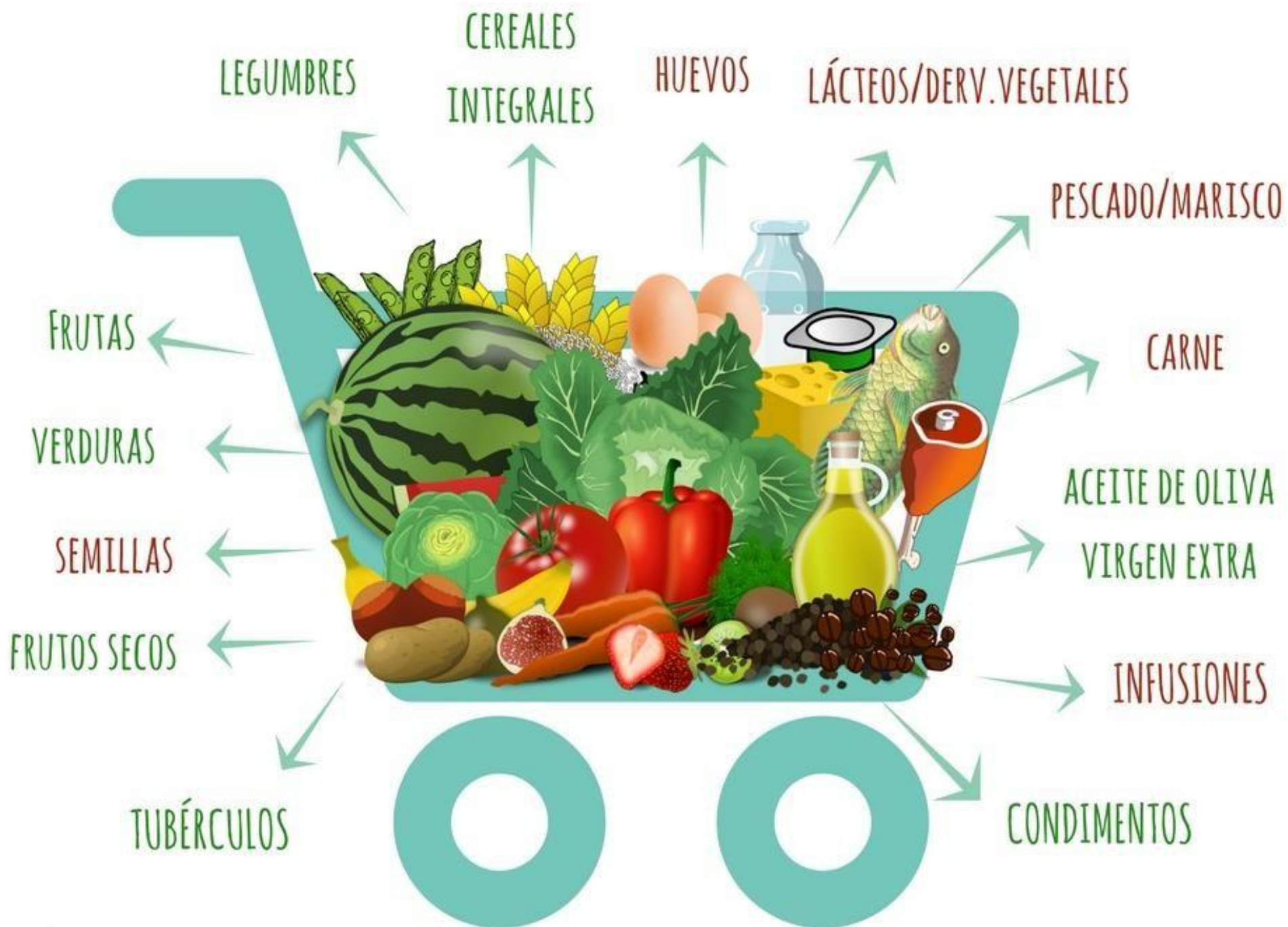

Small changes

TO EAT BETTER

More	*Change to*	*Less*
FRUITS AND VEGETABLE	WATER	SALT
LEGUMES	WHOLE FOODS	SUGARS
NUTS	VIRGIN OLIVE OIL	RED AND PROCESSED MEAT
ACTIVE AND SOCIAL LIFE	SEASONAL AND LOCAL FOODS	ULTRA-PROCESSED FOOD

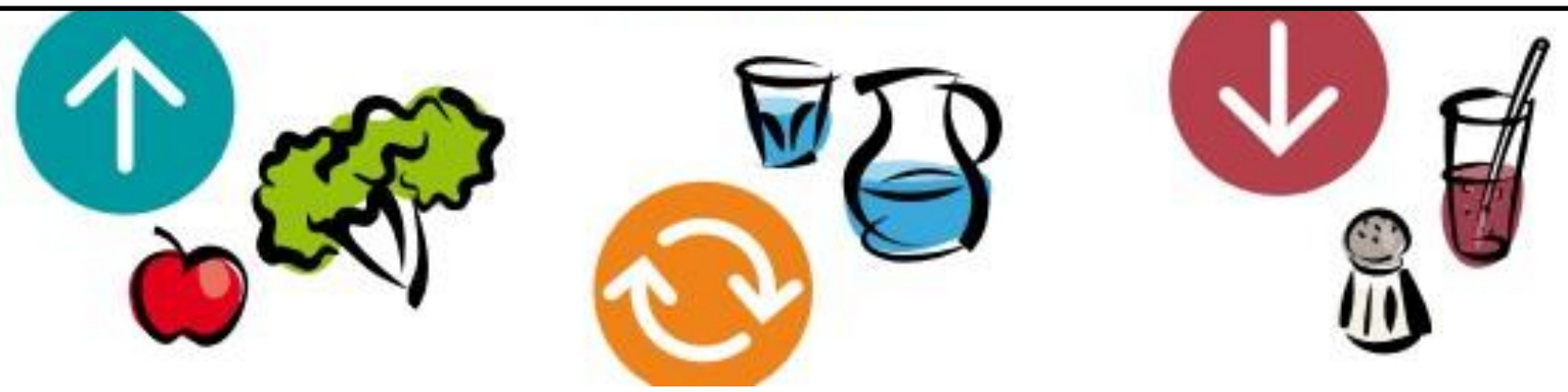

¡REMEMBER!

FRUIT AND A DAILY ROUTINE FOR LIVING TO BE A CENTENARIAN

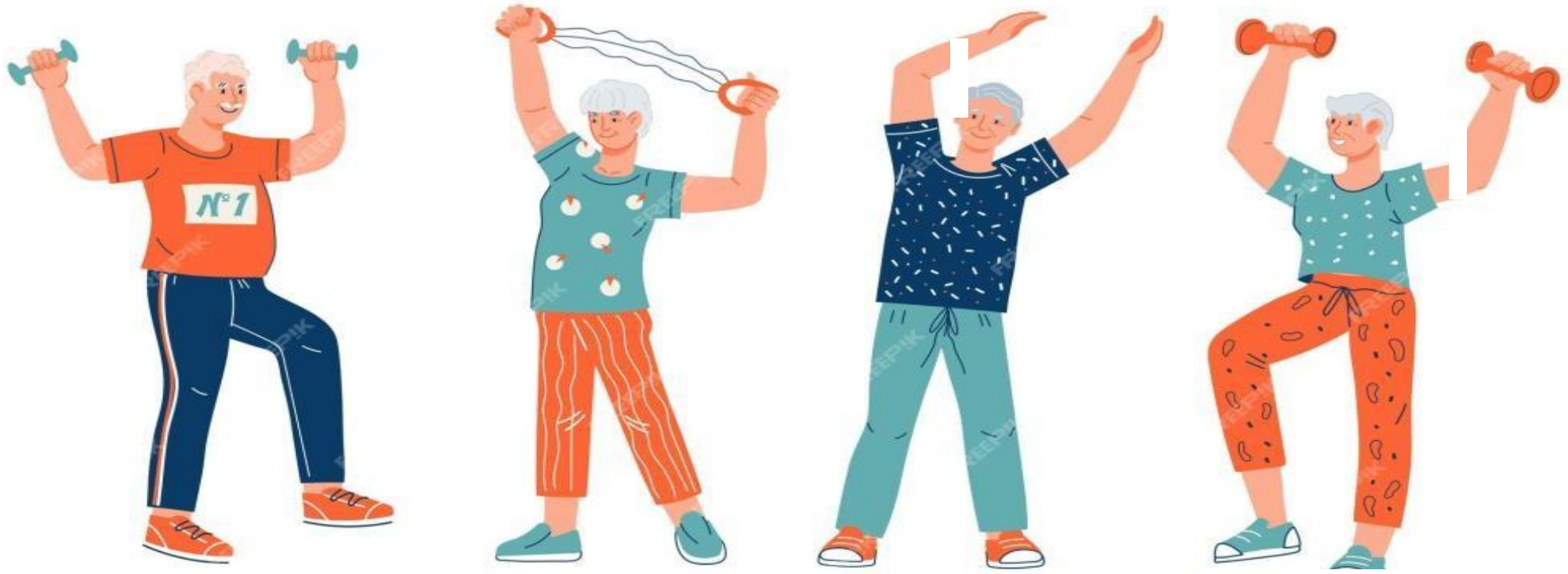

VNiVERSiDAD Ð SALAMANCA

Ana Mª Murillo Zaldívar

Ayuntamiento de Salamanca

LEARNING TO UNDERSTAND FOOD LABELS

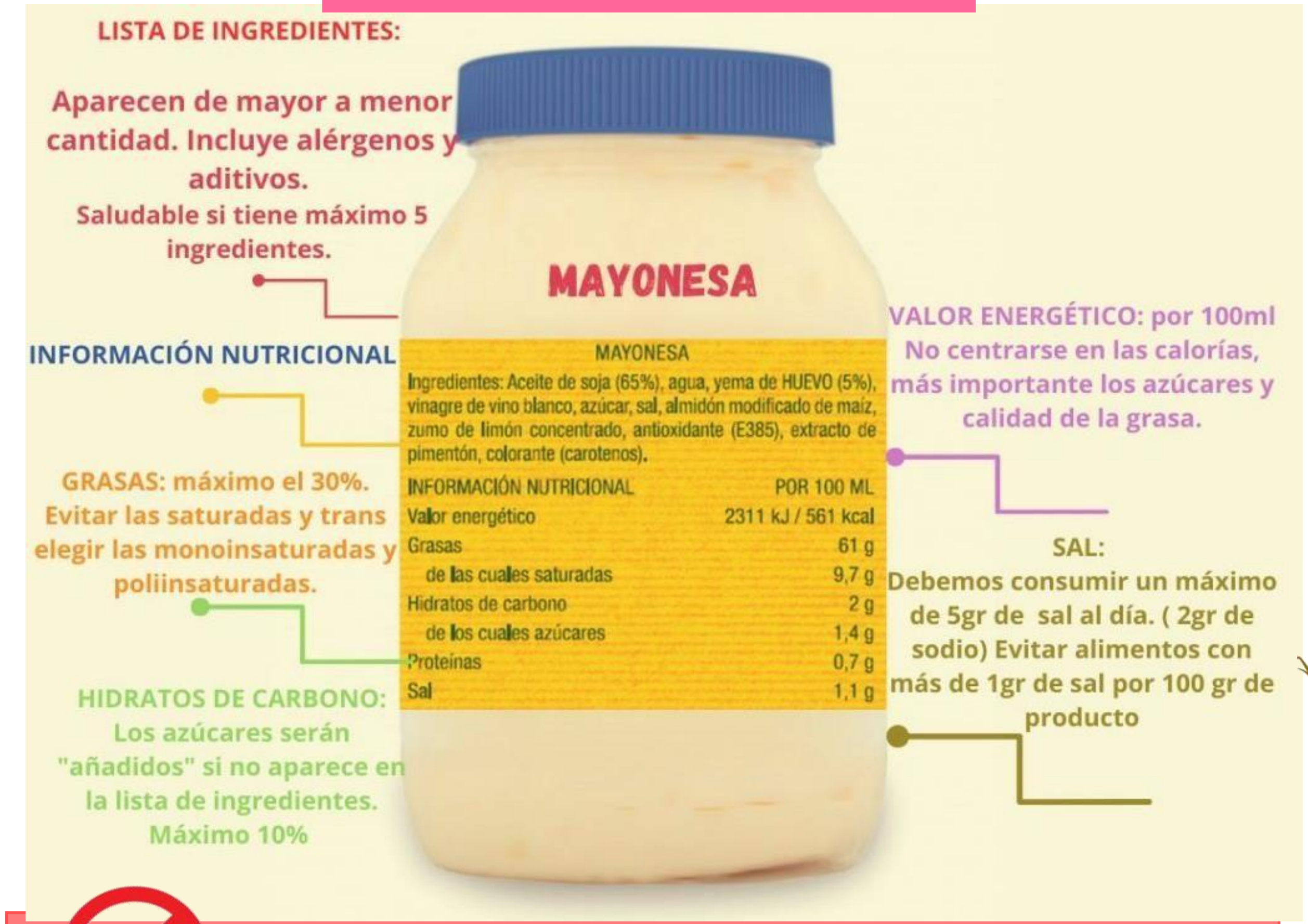

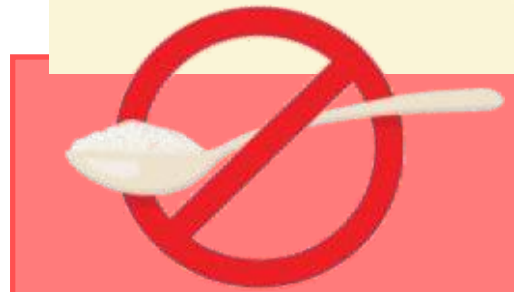

HOW TO SPOT ADDED SUGAR – DON'T BE FOOLED

Most processed and packaged foods contain added sugar, sowhen we eat them we are consuming 'hidden sugar', which is difficultto identify. The key to knowing whether a food contains added sugar isto look at the nutritional information on the label, specifically the section that says 'carbohydrates,of which sugars', and the list of ingredients.

- In the list of ingredients, added sugar can be identified as "sugar", andas glucose, sucrose, dextrose, glucose syrup, fructose, oligofructose,fructose syrup, caramel, honey, concentrated fruit juice, dextrin, maltdextrin, modified maize starch, tapioca…

PACKAGED FOOD

It is important to check the percentage of the main ingredient. For example, in the case of sliced ham, it is considered healthy if the percentage of ham or turkey is HIGHER than 85% or 90%.

THE IMPORTANCE OF FIBRE IN THE DIET

Fibre is found exclusively in plant-based foods:fruit, vegetables, cereals, pulses and nuts.

Recommendations:

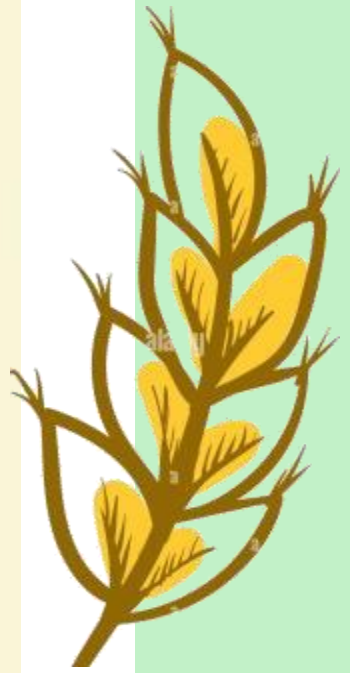

- Eat 2–3 portions of vegetables a day, either raw or cooked.
- Eat 2–3 pieces of fruit every day: whole, raw and with the skin on if possible, avoiding juices as they are low in fibre.
- Eat pulses at least twice a week.
- Choose whole meal cereals and flours.
- Eat nuts at least three times a week.

Benefits of fibre:

- Lowers bad cholesterol and reduces the risk ofcardiovascular disease.
- Helps control blood sugar levels and promotes a feeling of fullness,thereby reducing the risk of obesity.

The advice is to buy fresh, locally sourced food (fruit, vegetables, greens, potatoes, bread, meat, fish, etc.) and to limit processed foods as much as possible.

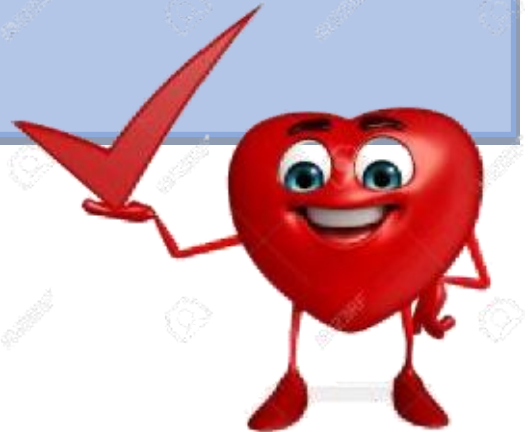

Supplement: Supplementary file 2 [file Data_Sheet_2.pdf]
